# Subarachnoid Transplant of the Human Neuronal hNT2.19 Serotonergic Cell Line Attenuates Behavioral Hypersensitivity without Affecting Motor Dysfunction after Severe Contusive Spinal Cord Injury

**DOI:** 10.1155/2011/891605

**Published:** 2011-06-01

**Authors:** Mary J. Eaton, Eva Widerström-Noga, Stacey Quintero Wolfe

**Affiliations:** ^1^Miami VA Health System Center, D806C, 1201 NW 16th Street, Miami, FL 33125, USA; ^2^The Miami Project to Cure Paralysis, Miller School of Medicine, University of Miami, 1095 NW 14th Terrace, Miami, FL 33136, USA; ^3^Department of Neurosurgery, Tripler Army Medical Center, 1 Jarrett White Road, Honolulu, HI 96859-5000, USA

## Abstract

Transplant of cells which make biologic agents that can modulate the sensory and motor responses after spinal cord injury (SCI) would be useful to treat pain and paralysis. To address this need for clinically useful human cells, a unique neuronal cell line that synthesizes and secretes/releases the neurotransmitter serotonin (5HT) was isolated. Hind paw tactile allodynia and thermal hyperalgesia induced by severe contusive SCI were potently reversed after lumbar subarachnoid transplant of differentiated cells, but had no effect on open field motor scores, stride length, foot rotation, base of support, or gridwalk footfall errors associated with the SCI. The sensory effects appeared 1 week after transplant and did not diminish during the 8-week course of the experiment when grafts were placed 2 weeks after SCI. Many grafted cells were still present and synthesizing 5HT at the end of the study. These data suggest that the human neuronal serotonergic hNT2.19 cells can be used as a biologic minipump for receiving SCI-related neuropathic pain, but likely requires intraspinal grafts for motor recovery.

## 1. Introduction

Current understanding of central and supraspinal [1] mechanisms for the induction and maintenance of chronic pain after SCI suggests a major role for the hypofunction of serotonergic (5HT) inhibitory systems [2–4]. SCI also leads to the loss of descending serotonergic excitatory inputs caudal to the lesion site and altered neurotransmitter levels within the ventral horn *α*-motoneurons, which contributes to motor dysfunction [5, 6]. Multiple animal studies have used a 5HT rat cell line [5, 7–9] or 5HT raphe transplants [10, 11] as a means to ameliorate some of the impairments associated with spinal injury. Supplemental cell therapy after spinal injury can create a spinal environment conducive to the amelioration of local damage and promotion of a regenerative response in multiple axonal populations, including descending spinal serotonin fibers [12] or reverse neuropathic pain by reversing hyperexcitability in the dorsal horn [9]. Thus, a human 5HT neuronal cell line that can restore the function(s) of a damaged nervous system, and be genetically manipulated, stored, and expanded, would potentially be extremely useful for clinical applications. 

A number of animal models have been developed for SCI to produce reliable and consistent conditions mimicking human neuropathic pain. These include photochemically induced ischemia [13], hemisection of the spinal cord [14, 15], and excitotoxic lesions using intraspinal injections of excitatory amino acid agonists [16–18]. In addition, the severe contusive SCI model with a weight drop device (NYU impact injury) has been used to examine both pain [19, 20] and motor dysfunction [21–23] in a variety of studies. These models induce changes in intraspinal biochemistry through the loss, among other mechanisms, of modulation by the 5HT-releasing interneurons in the cord and a loss of supraspinal control of voluntary locomotor activity. These mechanisms are further supported by the studies of denervation supersensitivity to 5-HT following SCI, which corroborate behavioral studies showing the effectiveness of 5-HT in reducing allodynia and hyperalgesia after SCI [4] and improvement of motor function with 5HT1A receptor agonists [24]. Previous studies of locomotor recovery after SCI have used intraspinal transplants of hNT2.19 cells, an immortalized human neuronal cell line which actively secretes serotonin, to enhance 5-HT levels near lumbar motor pools [23] and to partially recover locomotor function in the nude rat following severe contusive SCI. The neurotransmitter 5HT is naturally present in the dorsal and ventral horns of the spinal cord and in spinal pathways mediating nociceptive and motor function. However, 5HT is not likely to be present in adequate amounts after nervous system injury to effectively modulate the sensory/motor imbalance that induces neuropathic pain and motor impairments. Replacement or supplementation of endogenous 5HT for sensory and motor recovery may be a reasonable approach, since its loss after SCI is dependent on injury severity [25] and correlates with loss of motor function [26] and the alterations in the sensory system that provide an environment conducive of neuropathic pain [27]. Unfortunately, pharmacologic modulation of 5HT is fraught with methodological problems that could be overcome or enhanced by a dependable supply of authentic 5HT produced by cellular minipumps located near (subarachnoid space) or in (ventral motor centers) the spinal cord, rather than 5HT-receptor agonists.

More than twenty years ago it was discovered that when treated with retinoic acid (RA), a human embryonic carcinoma cell line, NTera2cl.D/l (NT2), differentiates irreversibly into several morphologically and phenotypically distinct cell types, including terminally differentiated postmitotc CNS neurons [28]. Successive replating of RA-treated NT2 cells, in the presence of growth inhibitors, results in the isolation of purified human neurons [29], which have been extensively characterized and tested in vivo in a number of animal models of traumatic injury and neurodegenerative disease [28, 30]. Potential application of these progenitor NT2-derived neurons in cell transplantation therapy for CNS disorders has been demonstrated in Phase I-II clinical trials for the treatment of stroke [31] and can likely be utilized for further reparative transplant strategies.

One phenotype present within the NT2 parent population synthesizes the inhibitory neurotransmitter 5HT [32]. From the variety of phenotypes expressed after differentiation from NT2 cell line, we sought to subclone a human neural cell line from the NT2 heritage that was specific to the synthesis and secretion of 5HT, to characterize these cells in vitro and test their ability to affect nociceptive and motor function in a SCI-pain/motor model. We have previously described the use of 5HT cell therapy with a rat cell line that is able to consistently reverse neuropathic pain after a partial nerve injury [7] and hemisection SCI [33]. Here we expand on our previous investigations [23] with human hNT2.19 5HT-secreting cell grafts in the severe contusion SCI model of chronic pain and motor dysfunction and report findings with human neuronal 5HT cell therapy, where hNT2.19 subarachnoid grafts are able to significantly reduce behavioral hypersensitivity, but not motor dysfunction.

## 2. Materials and Methods

### 2.1. Development of the Human hNT2.19 and hNT2.6 Control Cell Lines

Human neuronal cell lines were subcloned from the parental NTera2cl.D/l (NT2) [34] cell line by serial dilution, isolation of single cells that form colonies, and analysis of multiple cell lines using a variety of immunohistochemical markers, including 5HT, to determine the differentiated neurotransmitter phenotype of the various cell lines. We took advantage of a rapid aggregation method [35] for retinoic acid treatment and differentiation into the human NT2-derived neuronal phenotype to select various cell lines, as reported previously [18, 23]. Although we derived a number of human NT2 neurotransmitter cell lines by these methods, we have used the specific hNT2.19 cell line for further characterization and transplant in severe contusive SCI pain. Additionally, a nonserotonergic sister cell line was isolated, named hNT2.6, and used as a negative control in transplant studies.

The rapid aggregation method [35] for retinoic acid treatment and differentiation was also used for the preparation of cultures of differentiated hNT2.19 and hNT2.6 cells in vitro for characterization and transplant. Briefly, proliferating cultures of hNT2.19 and hNT2.6 cells were grown to near confluence at 37°C in proliferation medium: Dulbecco's Modified Eagle Medium/Ham's F12 (DMEM/F12, Gibco)/ 10% fetal bovine serum (FBS, HyClone, Logan, Utah)/ 2 mM L-glutamine (Gibco) freshly added/1% Pen-Strep (P.S.; Gibco) with every 3rd day media change. When cells were near 100% confluent, they were replated to 100 mm Petri dish (VWR) in DMEM/high-glucose (HG)/10% FBS/10 *μ*M all-trans retinoic acid (RA) (Sigma)/15 mM HEPES, pH 8.0/2 mM L-glutamine/1%Pen-Strep and continued for two weeks, with fresh media changed every 2 days. After removal with 0.5 mM EDTA, centrifugation, and resuspension, cells were replated to 100 mm tissue culture dishes (Falcon) which had been coated with mouse laminin [(Biomedical Technologies, Stoughton, MA; 20 *μ*g/mL in DPBS)/poly-L-lysine (Sigma; 20 *μ*g/mL in PBS)]. They were then continued in DMEM/high-glucose (HG)/5% FBS/1% Pen-Strep (P.S.)/L-glutamine, 2 mM, at a pH of 7.4, for 9–24 hrs, before the addition of cytosine-D-arabinofuranoside (araC) (Sigma; 1 *μ*M), plus uridine (Sigma; 10 *μ*M), for nonneuronal growth inhibition. After seven days, cells were briefly exposed to warmed trypsin/0.5 mM EDTA and adherent surface cells removed with DMEM/HG/5% FBS/P.S./L-glutamine, 2 mM, at a pH of 7.4. These cells were centrifuged, resuspended, and replated on 60 mm tissue culture dishes (Falcon), coated with mouse laminin [(Biomedical Technologies, Inc; 20 *μ*g/mL in DPBS)/poly-L-lysine (Sigma; 20 *μ*g/mL)], and continued in DMEM-HG/5% FBS/P.S./L-glutamine, 2 mM at a pH of 7.4 at 37°C for two weeks before transplant, with media changed every 2-3 days.

### 2.2. Immunohistochemistry of hNT2.19 Cells In Vitro

Monoclonal antibody antibromodeoxyuridine (BrdU; #347580; dilution 1 : 10) was purchased from Becton-Dickson, San Jose, CA. Polyclonal antibody antifibroblast growth factor-4 (FGF-4; AF235; dilution 1 : 20) was purchased from R&D Systems, Minneapolis, MN. The polyclonal antibody anti-5HT (ab10385-50; dilution 1/100 (in vitro)) was purchased from Abcam Inc, Cambridge, MA. The polyclonal antibody anti-C-terminus of neurofilament high (NFH; AB1989; dilution 1 : 100) was purchased from Chemicon (Millipore), Billerica, MA. Polyclonal antibody antihuman neuron-specific enolase (hNSE; 17437; dilution 1 : 1000) was purchased from Polysciences, Warrington, PA. Monoclonal antibody anti-beta-tubulin III (TuJ1, MO15013; dilution 1 : 100) was purchased from Neuromics, Edina, MN. The monoclonal antibody antitransforming growth factor-alpha (TGF-*α*; ab9578; dilution 1 : 100) was purchased from Abcam Inc., Cambridge, MA. The monoclonal antibody antineurofilament light (NFL; MCA-DA2; dilution 1 : 50) was purchased from EnCor Biotechnology Inc, Alachua, FL. Monoclonal antibody anti-C-terminus of anti-neurofilament medium (NFM; MCA-3H11; dilution 1 : 50) was purchased from EnCor Biotechnology Inc, Alachua, FL. The hNT2-19 cells, after two weeks of RA treatment and mitotic inhibitors, were replated to differentiate in 8-well laminin/poly-L-lysine coated Permanox slides, and differentiation continued for various times before immunostaining. The cells were then fixed for 10 min at 4°C with 4% paraformaldehyde and 0.1% gluteraldehyde in 0.1 M phosphate buffer, pH 7.4. All immunohistochemistry experiments included the use of a negative control, substitution of specific primary antibody with species IgG, to insure that positive signal was specific for the antigen. *For the BrdU immunostaining*: after fixation and rinsing in PBS, pH 7.4 at room temperature, hNT2-19 cells were incubated with 2 N HCl for 20 min at room temperature, rinsed ×3 with PBS, incubated with borate buffer (pH 8.5)/0.01 M boric acid /0.5 M Na borate (1 : 1) for 15 min at room temperature, rinsed for three times with PBS, and then permeabilized for 30 min at room temperature with blocking buffer before incubation with the primary anti-BrdU antibody. *For all other *in vitro* immunostaining experiments*: after fixation and rinsing in PBS, pH 7.4 at room temperature, fixed hNT2-19 cells were permeabilized for 30 min at room temperature with 0.5% Triton X-100 in PBS in the presence of 5% normal goat serum (the blocking buffer), before the addition of the individual primary antibody, usually overnight at 4°C. The staining was completed by incubation with the specific antispecies IgG secondary conjugated to Alexa Fluor 488 Green (dilution 1 : 100), purchased from Molecular Probe, Eugene, OR, for two hours at room temperature. After staining, slides were cover-slipped using Vectashield mounting medium with DAPI (Vector Laboratories, Burlingame, CA). Photo images were taken with a Zeiss microscope (Axioplan II Metamorphosis program). All staining experiments were independently repeated at least ×3, to insure that micrographs are representative. 

### 2.3. HPLC Analysis of hNT2.19 and hNT2.6 Cells In Vitro

#### 2.3.1. 5HT and Catecholamines

In order to examine the 5HT and catecholamine content and release in differentiated hNT2.19 and hNT2.6 cells, cells were differentiated for 2 wks at 37°C after plating in 35 mm laminin/poly-L-lysine-coated 6-well plates. Cell numbers were determined in sister wells by trypan blue exclusion and counting. Either 5HT/catecholamine content (in cells) or 5HT/catecholamine release (into the media) was examined by HPLC to determine the content or basal or stimulated level of 5HT and catecholamine release into the media. For 5HT content, cells were collected into 1.5 mL centrifuge tube (in distilled water), cells broken by lysis with 0.05 N PCA (perchloric acid), tube contents centrifuged at 4°C, and supernatant collected for HPLC. Similar cell culture samples were also incubated with either normal K+ (2.95 mM) Krebs-Ringer buffer or high K+ (100 mM) buffer for 15 min at 37°C and the media collected to determine the levels of 5HT or catecholamine released into the media by membrane depolarization. The media samples were kept on ice and immediately analyzed by HPLC. The HPLC system consisted of a solvent-delivery pump (Waters 510 Pump), an autosampler (Waters 717 plus Autosampler), and an electrochemical detector (ESA Coulochem II); Electrode: ESA Microdialysis Cell 5014A (DC CH1: −150 mV, DC CH2 : 300 mV, 500 mA); Guard Cell Model 5020 (GC 350 mV). Elution was carried out at room temperature with a reversed-phase column (C18, 5 *μ*M, 150 × 3, BetaBasic-18, Thermo) and MDTM mobile phase (ESA Inc. 70-1332); it consisted of 75 mM of NaH_2_PO_4_, 1.7 mM of C_8_H_17_O_3_SNa, 100 *μ*L/L of TEA, 25 *μ*M of EDTA, 10% acetonitrile, pH 3.0 adjusted by H_3_PO_4_ at a flow rate of 0.6 mL/min. Ordinarily the norepinephrine appeared at about 2.3 min; the epinephrine at about 2.6 min; 5HT at about 7.5 min.

### 2.4. Animal Study Design

Once the hNT2.19 and hNT2.6 cell lines were characterized with an understanding of the neuronal phenotype and secretory properties, the effect of grafts of these cells on pain and motor behaviors after severe contusive SCI was studied. Adult female Sprague-Dawley rats (Harlan; approximately 200–250 grams) were used for all behavioral experiments. The rats were housed 2 per cage with rat chow and water ad lib on a 12/12 hr light/dark cycle. Rats were acclimated and pretrained to two behavioral sensory tests: tactile allodynia (hindpaw withdrawal from a normally innocuous mechanical stimulus) and thermal hyperalgesia (hindpaw withdrawal from a noxious heat source). Additionally, and on alternate days, all animals were examined for motor behaviors, which included the BBB open-field testing. These tests were performed weekly (but on different days to reduce animal stress) for the duration of the 60-day experiment. Additionally, before and at end of the experiment before euthanasia, all animals were examined for four other motor behavioral tests: gridwalk error, degree of hindlimb rotation, base of hindlimb support, and measurement of stride length. Following behavioral baseline measures before any surgery, the animals then underwent a severe contusive SCI (25 mm weight drop, NYU impactor) to induce behavioral hypersensitivity to tactile and thermal stimuli, as confirmed by a vigorous response to sensory behavioral testing, and developing permanent motor dysfunction. Two weeks after injury or laminectomy only, animals to be transplanted received either a lumbar intrathecal cell graft with either hNT2.19 cells (differentiated for 2 weeks in vitro before transplant) or negative-control hNT2.6 cells. A third group of animals served as a control group and received only the SCI but no transplant. A fourth group of animals received laminectomy alone, rather than the SCI, and served as surgery controls. Two additional groups of rats received laminectomies, rather than full SCI, and were transplanted at the two-week time point with either hNT2.19 or hNT2.6 cells. All groups of animals received Cyclosporine A (CsA) immunosuppression (i.p.,10 mg/kg, daily) at the time points corresponding to 1 day prior to and 13 days following transplant. The animals were sacrificed after eight weeks of behavioral testing and examined for the presence of surviving grafted hNT2.19 or hNT2.6 cells.

All surgical interventions, pre- and postsurgical animal care, and euthanasia were in accordance with the Laboratory Animal Welfare Act, *Guide for the Care and Use of Laboratory Animals* (NIH, DHEW Pub. no. 78-23, Revised, 1978) and guidelines provided by the Animal Care and Use Committee of the Veteran's Association Medical Center (VAMC), Miami, Fl. All behavioral testing was performed under blinded conditions to eliminate experimental bias and data analyzed and unblinded by the statistician at the end of the experiment. Each specific intervention or test is described in detail below. 

### 2.5. Contusive Spinal Cord Injury

Contusion injury was induced by the weight drop device developed at New York University [22]. Adult female Fischer rats (Harlan, *n* ≥ 5; 200–250 g) were housed according to NIH and USDA guidelines. The Institutional Animal Care and Use Committee of the Miami VAMC approved all animal procedures. Animals were anesthetized using an IP injection of a mixture of ketamine (35 mg/Kg) and xylazine (5 mg/Kg), all 0.65 mL/Kg, and then placed on a surgical table on a heating pad (37 ± 0.5°C) with pedal and eye blink reflexes assessed for deep anesthesia before beginning procedures. The back region was shaved and aseptically prepared with betadine. Lacrilube ophthalmic ointment (Allergan Pharmaceuticals, Irvine, CA) was applied to the eyes to prevent drying and bicillin (0.02 mL/100 mg body weight, 300 U/mL; J. Buck, Inc., Owings Mills, MO) administered intramuscularly. Following anesthesia, a vertical incision was made along the thoracic vertebra and the superficial muscle and skin retracted. A laminectomy performed at thoracic vertebra T7 exposed the dorsal surface of the spinal cord underneath (T8) without disrupting the dura mater. Stabilization clamps were placed around the vertebrae at T6 and T12 to support the column during impact. The exposed spinal cord was severely injured by dropping a 10.0 g rod from a height of 25.0 mm. The contusion impact velocity and compression were monitored to guarantee consistency between animals. After injury, the muscles were sutured in layers and the skin closed with absorbable sutures (Ethicon, Inc). The rats were allowed to recover in a warmed cage with water and food easily accessible. Bicillin (0.02 mL/100 mg body weight, 300 U/mL, i.m.) was administered 2, 4, and 6d after the contusion injury. The rats were maintained for 8 wks after injury, including gentle twice daily manual bladder expression to prevent the development of cystitis.

### 2.6. Cell Culture and Transplant of hNT2.19 and hNT2.6 Cells

The hNT2.19 5HT and control hNT2.6 cells that had been predifferentiated (as above) for 2 weeks in vitro were prepared for transplant studies. Briefly, cells were rinsed with warmed Cellstripper (Voigt Global Dist.), media replaced with another 3 mL of Cellstripper for one minute, and then rinsed with warmed Hank's buffered salt solution (HBSS) for complete cell removal from the TC plate. Viability and cell counts were assessed by trypan blue exclusion, and the cells were suspended in 10^0^ *μ*l of Ca^2+^-Mg^2+^-free Hank's buffered saline solution (CMF-HBSS). An aliquot of one million cells (1 × 10^6^ cells/10 *μ*L buffer) was prepared immediately prior to each transplant to assure near 100% viability at the beginning of the experiment; grafting was within 30 min of cell preparation.

The animals to be transplanted, one day after showing a vigorous response to behavioral testing, were anesthetized with a mixture of ketamine, xylazine, and acepromazine (0.65 mL/kg). For subarachnoid grafts, the previous laminectomy site (T7) was exposed and a small dural and arachnoidal incision was made and a 2-3 mm segment of polyethylene (PE-10) tubing, connected to a micropipette, inserted through the durotomy in a caudal direction. The one million cells (hNT2.19 or hNT2.6) were injected into the intrathecal space at spinal segment L1–L3 and the fascia and skin closed. Again, no additional analgesia was used. The animals were allowed to recover at 37°C for 12 hrs, after which time they were returned to the animal care facility. All rats, including those not provided cell transplants, received immunosuppressive therapy with CsA, injected i.p., which began one day before cell transplant and continued daily for 13 days.

## 3. Behavioral Testing

### 3.1. Thermal Hyperalgesia Testing

Methods for testing thermal hyperalgesia with a Hargreaves device have been described elsewhere [36]. Animals were placed in a clear plexiglass box on an elevated plexiglass floor. Animals were allowed to acclimate for approximately 5 min. A constant intensity, radiant heat source was aimed at the midplantar area of the hind paws. The time, in seconds, from initial heat source activation until paw withdrawal, was recorded. Five minutes were allowed between assessments. Three to four latency measurements for each paw were recorded and the mean and standard error of the mean (SEM) calculated for each hindpaw. Animals were tested 3 times, one week apart, for 2 wks prior to the injury (baseline) and then weekly for the duration of the experiment. In order to provide a robust baseline value for comparison purposes, baseline data was averaged to a mean baseline based on the three baseline tests.

### 3.2. Tactile Allodynia Testing

Mechanical allodynia, the occurrence of foot withdrawal in response to normally innocuous mechanical stimuli, was tested using an automated, electronic von Frey anesthesiometer (IITC, Inc) [37]. Animals were placed in a plexiglass box with an elevated mesh floor. After the animal was acclimated for 5 min, the device tip was applied perpendicular to the midplantar area of each hindpaw and depressed slowly until the animal withdrew the paw from pressure. The value, in grams, was recorded for each of the 3 trials. A single trial of stimuli consisted of three to four applications of the von Frey tip within a 10-second period, to ensure a consistent response. The values obtained for each hindpaw were averaged and the SEM calculated. The animals were tested 3 times, one week apart, for 2-3 wks prior to the injury (baseline) and then weekly for the duration of the experiment. In order to provide a robust baseline value for comparison purposes, all baseline data was averaged to a mean baseline based on the three baseline tests.

### 3.3. BBB Motor Behavior Testing

Two wks prior to the injury, open-field locomotor functions of all animals were assessed using the Basso, Beattie, and Bresnahan (BBB) locomotor rating scale [38]. Behavioral assessments were then performed on days 1 and 7 following the injury and weekly thereafter. The BBB score was used to study the functional recovery stages following the injury, by categorizing the rat hindlimb movements, trunk position and stability, coordination, stepping and paw placement and tail position. Rats were placed in a small, shallow, empty children's swimming pool and allowed to move freely for 60 mins of exercise, during which their motor behaviors were observed and scored according to the BBB scale. All observations were made by at least two independent observers, who were unaware of the extent or nature of the injury. The animals were rated on a scale of 0 to 21.

### 3.4. Footprint Analysis and Gridwalk Footfall Error

Footprint analysis was performed before and at 8 weeks postinjury using a modified protocol by de Medinaceli et al. [39]. The animal's fore and hind paws were inked with different colors to record footprints on paper that covered a narrow runway of 1 m in length and 7 cm in width. A series of at least eight sequential steps were used to determine the mean values for each measurement of limb rotation, stride length, and base of support. The base of support was determined by measuring the core-to-core distance of the central pads of the paws. The limb rotation was defined by the angle formed by the intersection of the line through the print of the third digit and the line through the central pad parallel to the walking direction. Stride length was measured between the central pads of two consecutive prints on each side. For the gridwalk test, deficits in descending fine motor control was examined at 8 weeks postinjury by assessing the ability to navigate across a 1 m long runway with irregularly assigned gaps (0.5–5.0 cm) between round metal bars, as described previously by Metz et al. [40]. Crossing this runway required that animals accurately place their limbs on the bars. In baseline training and postinjury testing, every animal crossed the grid at least three times. The numbers of footfalls (errors) for hindlimbs were counted in each crossing, and a mean error rate was calculated.

### 3.5. Immunohistochemistry In Vivo

For immunohistochemistry of sectioned spinal cord tissues, the polyclonal antibody anti-5HT (ab10385; dilution 1/100 (in vivo)) was purchased from Abcam Inc, Cambridge, MA, and the antihuman TuJ1 antibody (Neuron-specific class III beta-tubulin) was purchased from Neuromics, Edina, MN (MO15013; dilution 1/100 (in vivo).

### 3.6. Fixation

Spinal cords were fixed to examine cell graft survival and 5HT and TuJ1 staining, 8 weeks after contusive SCI (6 weeks after transplant). Transcardial perfusion with 4% paraformaldehyde [41] and 0.1% glutraldehyde was performed. Rats were euthanized for tissue fixation by a combination of pentobarbital overdose and exsanguination. Animals were anesthetized with an interperitoneal injection of sodium pentobarbital (12 mg/100 g). Once the appropriate level of anesthesia was reached (i.e., no corneal or withdrawal reflexes), the rat was transcardially perfused with the aldehydes. After perfusion, the spinal cord, including transplant, was removed and histologically processed. After removal from the vertebral column, cords were stored in fix for 12 hrs, 4°C. The cords were cryoprotected by equilibration in 30% sucrose and PBS overnight, 4°C and then frozen and stored at −80°C. Cords were embedded in Shandon M-1 Embedding Matrix (Thermo Electron Corp.) and sagittally cut in sequential 20 *μ*m sections with a Cryostat (Leica 1900). They were collected on noncoated slides (micro Slides, Snowcoat X-tra (Surgipath)). The slides were stored in a −20°C freezer and removed for defrosting before the immunostaining procedures. Every second section was stained for the human marker TuJ1 or 5HT and dehydrated, cleared, and mounted in Cytoseal 60 (Richard-Allan Scientific) after antibody staining. Processed slides were observed and photographed with a Nikon Digital Imagining Eclipse 90i Research Microscope.

### 3.7. TuJ1 Staining

Modified methods for staining spinal cord sections for the human neuron-specific class III beta-tubulin (TuJ1) to identify grafted hNT2.19 and hNT2.6 neurons after grafting have previously been described [42]. The sections were washed with 0.1 M PBS pH 7.4 and permeabilized with 0.4% Triton-X-100 in 0.1 M PBS, 10% normal goat serum (NGS) for one-hour. The sections were then incubated overnight at 4°C in the primary anti-TuJ1 antibody (1/100 DPBS), and the permeabilizing solution, followed by a one hour incubation at room temperature with the secondary antibody solution, biotinylated mouse IgG raised in goat (Vector; 1/200), a Peroxidase ABC reporter in 0.1 M PBS (Vector), and “VIP” substrate (Vector). Some sections were stained in the absence of primary antibody, and served as the negative controls.

### 3.8. 5HT Staining

Methods for staining lumbar spinal cord sections for 5HT and grafted hNT2-derived cell lines have been adapted from methods described elsewhere [23]. Sections were incubated with the primary antibody 5HT (1/100) with 0.4% Triton-X-100 in 0.1 M PBS and 10% NGS overnight at 4°C, followed by a one-hour incubation at room temperature with the secondary antibody solution, biotinylated anti-rabbit IgG (H + L), made in goat (Vector; 1/200) in 0.4% Triton-X-100 in 0.1 M PBS and 10% normal goat serum (NGS), a Peroxidase ABC reporter in 0.1 M PBS (Vector), and “VIP” substrate (Vector). Some sections were stained in the absence of primary antibody, and served as the negative controls.

### 3.9. Statistical Analysis

Statistical analyses were performed with PASW 17.0 for Windows. To determine differences between the groups and between time points, we used one-way analysis of variances (ANOVAs) and paired Student' *t*-tests. All *t*-tests were two tailed, and we used Bonferroni correction to adjust for multiple comparisons. A *P* value of  .05 or less was considered statistically significant.

## 4. Results

### 4.1. hNT2.19 Cell Line Characterization In Vitro

#### 4.1.1. The hNT2.19 Cell Line Has a Neuron-Like Morphology during Differentiation over Time In Vitro

**Figure 1:**
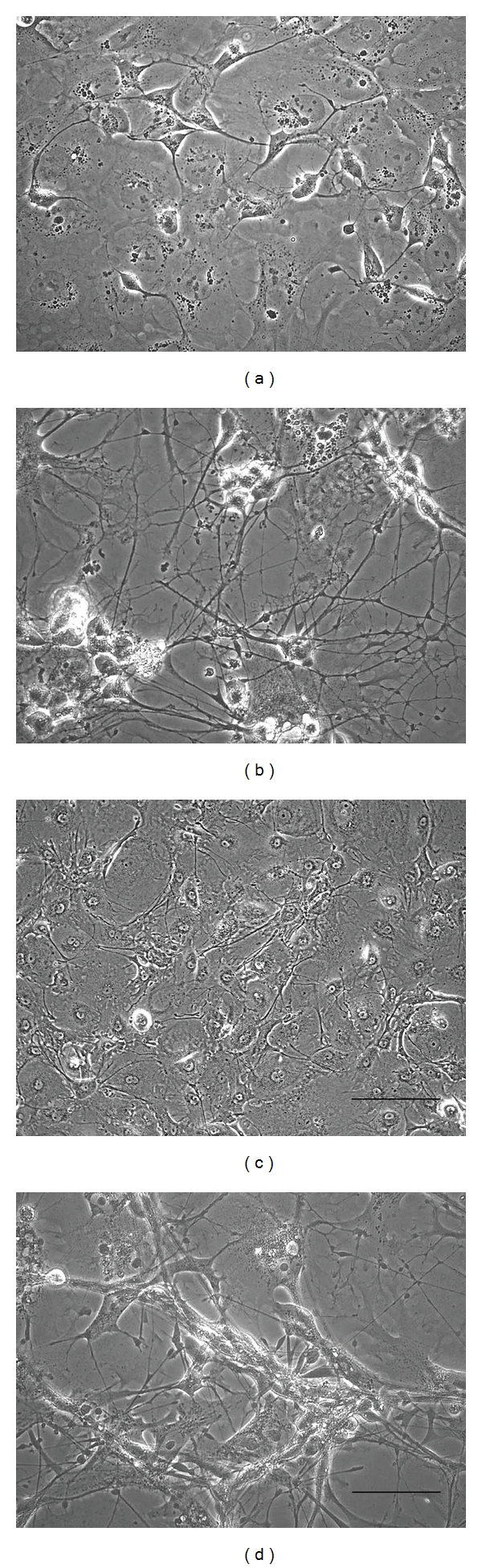
Morphology of the differentiation of the hNT2.19 and hNT2.6 cell lines in vitro. The hNT2.19 and hNT2.6 cell lines were treated for two weeks with retinoic acid (RA) and mitotic inhibitors and lifted to substrate-coated 8-well plastic tissue culture (TC) slides for differentiation and phase microscopy. As soon as 1 day of differentiation in culture under these conditions (a), hNT2.19 cells extend multipolar fibers, which are clearly visible at three days (b). The hNT2.19 cells continue to extend multiple fibers, forming a dense fiber network by 2 wks (c). The negative control hNT2.6 cells appear quite similar at 2 wks of differentiation (d). Magnification bar = 10 nm, (c); magnification bar = 20 nm, (a, b, d).

Once the hNT2.17 cells begin differentiation, after treatment with retinoic acid and mitotic inhibitors in vitro, they can easily be transferred to substrate-coated surfaces. When examined by phase microscopy during differentiation (Figure 1), the cells appear to have extended multipolar neuron-like processes as soon as one day in vitro (Figure 1(a)). By seven days, cells have begun to form dense fibers networks (Figure 1(b)). Within two weeks (Figure 1(c)), the cells continue to extend long fibers, but aggregate as balls of cells, eventually forming dense networks of fibers extending from the balled cells. The control hNT2.6 cells, here seen at 2 weeks of differentiation (Figure 1(d)), are nearly indistinguishable from the hNT2.19 cells. Both cell lines have been kept as long as 50 days of differentiation in culture, forming very dense fiber networks that cover the plate surface.

#### 4.1.2. The hNT2.19 Cells Incorporate BrdU with Proliferation but Not Differentiation In Vitro

**Figure 2:**
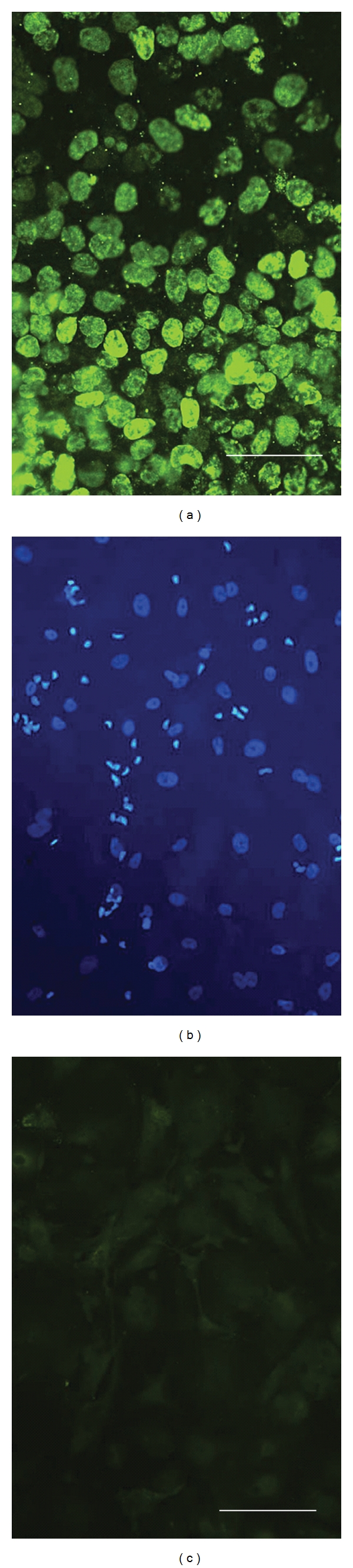
The BrdU signal in proliferating versus differentiating hNT2.19 cells in vitro. The hNT2.19 cells were either exposed to 1 *μ*M BrdU during 3 days of proliferation (a) or for 1 week during differentiation (b, c) in vitro. With an antibody directed against BrdU, proliferating cells incorporate abundant BrdU during proliferation (a). Viable differentiated cells were co-labeled with a DAPI stain (b), while the same field of differentiated cells did not incorporate any BrdU during differentiation (c). After two weeks of retinoic acid and mitotic inhibitors, the hNT2.19 cells cease dividing during differentiation, and did not incorporate BrdU. Magnification bar = 20 nm, (a); magnification bar = 30 nm, (b, c).

Bromodeoxyuridine (BrdU) immunostaining has been used a marker for proliferating cells in vitro [43] and in vivo [44], since dividing cells incorporate BrdU-labeled uridine into newly made DNA. The hNT2.19 cells were exposed to 1 mM BrdU in vitro during either proliferation or during differentiation before anti-BrdU immunostaining. Following 3 days of proliferation in the presence of BrDU (Figure 2(a)), the BrdU signal is intense and found in all the dividing cells. After one week of BrdU exposure during the first week of differentiation, hNT2.19 cells remained viable, as evidenced by DAPI staining (Figure 2(b)). The same field of differentiated hNT2.19 cells showed no anti-BrdU signal (Figure 2(c)).

#### 4.1.3. Differentiated hNT2.19 Cells Cease to Express Markers of Tumorgenicity with Differentiation In Vitro 

**Figure 3:**
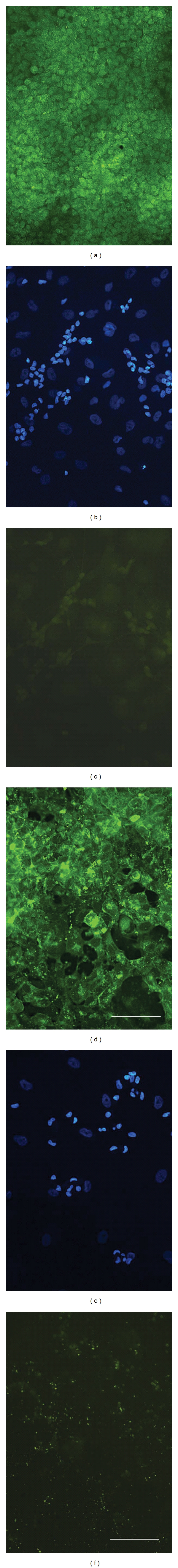
The tumor markers, TGF-*α* and FGF-4, in differentiating and proliferating hNT2.19 cells in vitro. In other sister cultures, the hNT2.19 cell line was either proliferated and stained for TGF-*α* (a) or treated for two weeks with RA and mitotic inhibitors and differentiated for one week (b, c) before TGF-*α* staining. Viable cells were located with DAPI stain (b). The same field of differentiated cells expressed no detectible TGF-*α* (c), while the signal was abundant in proliferating cells (a). In another set of sister cultures, the hNT2.19 cell line was either proliferated and stained for FGF-4 (d) or treated for two weeks with RA and mitotic inhibitors, and differentiated for one week (e, f). Viable cells were located with DAPI stain (e). The same field of differentiated cells expressed no detectible FGF-4 (f), while the signal was abundant in proliferating cells (d). Differentiated hNT2.19 cells do not express the tumor markers FGF-*α* or TGF-4. Magnification bar = 20 nm, (d); magnification bar = 30 nm, (a, b, c, e, f).

The question of possible tumorgenicity in the eventual clinical use of any differentiated cells is relevant to their characterization in vitro and in vivo [45]. Two important tumor markers, TGF-*α* and TGF-4, are associated with human embryonic carcinoma (EC) and NT2 cells. The parental NT2 cells are classified as EC cells because of their testicular germ cell origin and that they express the same cell-surface antigens during proliferation. Exposure of NT2 (proliferating) cells to retinoic acid results in postmitotic hNT2 cells, which do not form tumors or revert to a neoplastic state with transplantation [46]. A similar NT2-derived cell line, hNT2.17, has been characterized in vitro and in vivo and does not express tumor markers with differentiation in vitro or form tumors after transplant [18]. Undifferentiated NT2 cells express the protein TGF-*α*, which is involved in stimulation of cell proliferation [47], which decreases after RA treatment. Undifferentiated NT2 cells also express the protein FGF-4, which is abundant in a subset of germ cell cancers and promotes malignant growth of cultured ECs. Like TGF-*α*, it is repressed in NT2 cells after RA treatment [48]. When hNT2.19 cells are treated with RA and mitotic inhibitors, and differentiated in vitro, they cease to express both TGF-*α* and FGF-4. Proliferating hNT2.19 cells express abundant TGF-*α* and FGF-4 (Figures 3(a) and 3(d), resp.). After one week of differentiation, viable hNT2.19 cells (Figures 3(b) and 3(e), DAPI-stained) express no detectible TGF-*α* (Figure 3(c)) or FGF-4 (Figure 3(f)), both compared to viable DAPI-stained wells), suggesting they are no longer tumorigenic in vitro following RA treatment, mitotic inhibitors, and differentiation.

#### 4.1.4. The hNT2.19 Cells Express Human and Neural Markers with Differentiation

**Figure 4:**
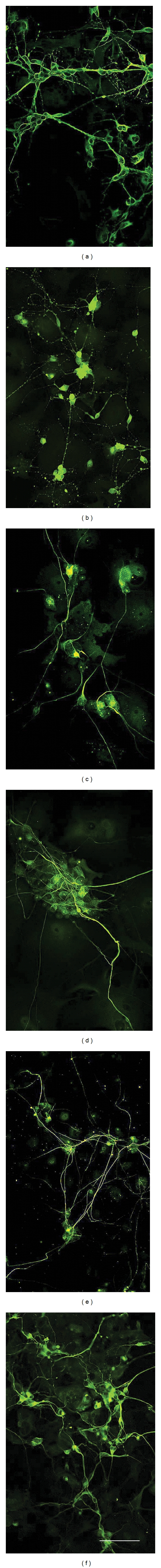
Neural and human markers with differentiation of the hNT2.19 cell line in vitro. The hNT2.19 cell line was treated for two weeks with retinoic acid and mitotic inhibitors and lifted to substrate-coated 8-well plastic TC slides for differentiation and immunohistochemistry for neuron-specific markers. As soon as 4 days in vitro, a variety of neural markers appeared, which remained strong until at least 6 wks of differentiation: TuJ1 (a), hNSE (b), NFL (c), NFM (d), and NFH (e). For comparison, the negative control hNT2.6 cell line was cultured similarly as the hNT2.19 cells and is here stained for TuJ1 (f). Magnification bar = 20 nm, (a–f).

Critical to the identity of the differentiated hNT2.19 cells is that they are exclusively neurons. Immunostaining with glial fibrillary acidic protein or vimentin antibodies did not result in a glial or proliferating precursor signal in hNT2.19 cells during differentiation (data not shown), features also demonstrated in the NT2 parent cell line [28] and the similar NT2-derived hNT2.17 cell line [18]. However, various neuron-specific markers were present as soon as 4 days of differentiation: TuJ1 (human neuron specific beta III tubulin protein), in hNT2.19 (Figure 4(a)) and hNT2.6 (Figure 4(f)) cells, human NSE (Figure 4(b)), NFL (Figure 4(c)), NFM (Figure 4(d)), and NFH (Figure 4(e)). These stained intensely until at least 6 wks of differentiation in vitro. TuJ1 has been commonly used to identify human neuronal cells in vitro and in vivo [42].

#### 4.1.5. The hNT2.19 Cell Line Expresses a Serotonin Neurotransmitter Phenotype with Differentiation

**Figure 5:**
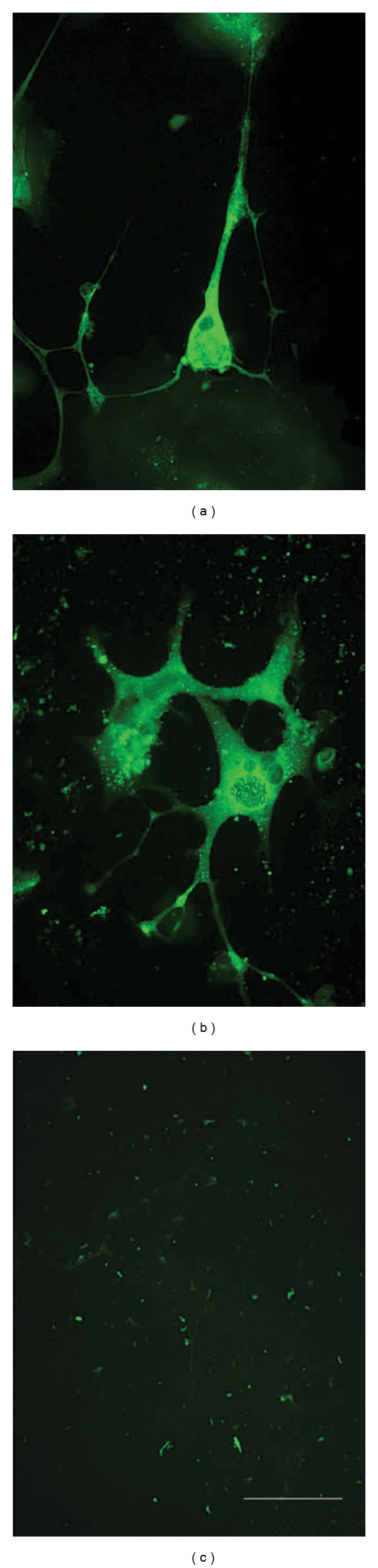
The hNT2.19 cell line expresses a 5HT phenotype with differentiation in vitro. The hNT2.19 cell line was treated for two weeks with retinoic acid and mitotic inhibitors and lifted to substrate-coated 8-well plastic TC slides for differentiation and immunohistochemistry for 5HT. All the hNT2.19 cells stain very brightly for the neurotransmitter 5HT (a, b). Both the cell soma and extending fibers contain a strong 5HT signal. As the fibers extend during differentiation, the fiber 5HT signal becomes concentrated, punctate-like, in bouton-like structures, as early as 2 wks in vitro (b). The negative control hNT2.6 cell line is seen in (c) after an anti-5HT immunostain; no 5HT signal is seen. Magnification bar = 20 nm, (a–c).

Easily observed during the early differentiation period with an antibody stain for 5HT, all the hNT2.19 cells stain for the neurotransmitter 5HT (Figures 5(a) and 5(b)). Both the cell soma and extending fibers contain a strong 5HT signal. As the fibers extend during differentiation, the fiber 5HT signal becomes concentrated, punctate-like, in bouton-like structures. Any similar immunostaining for 5HT in hNT2.6 cells (Figure 5(c)) was not detectable.

The hNT2.19 staining for other neurotransmitter phenotypes was negative, with no signals seen for choline acetyltransferase (ChAT), responsible for acetylcholine synthesis; tyrosine hydroxylase (TH), the rate-limiting enzyme for catecholamine synthesis; dopamine beta hydroxylase (DBH), which converts dopamine to norepinephrine; phenylethanolamine-methyltransferase (PNMT), which converts norepinephrine to epinephrine; calcitonin gene related peptide (CGRP); galanin; substance P; gamma aminobutyric acid (GABA); glycine; NMDA receptor 1 (NMDAR1); or chromagranin markers.

#### 4.1.6. HPLC for Serotonin (5HT) and Norepinephrine (Norepi) Synthesis and Release of Neurotransmitters in hNT2.19 and hNT2.6 Cells

**Figure 6:**
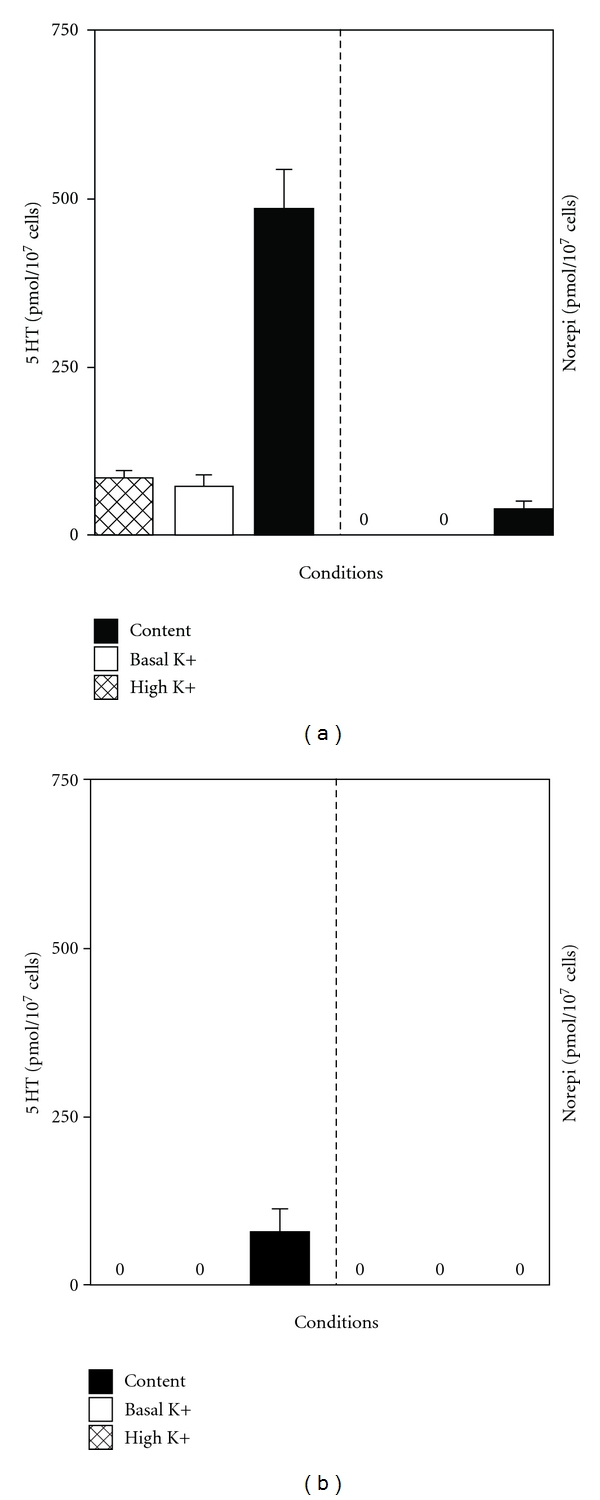
HPLC analysis: 5HT and norepinephrine content (synthesis), secretion, and release in hNT2.19 and hNT2.6 cells in vitro. The hNT2.19 (a) and the hNT2.6 (b) cell lines were differentiated, after RA and mitotic inhibitor treatment, for two weeks in 6-well substrate-coated plates before cell lysis and examination of cell content for 5HT or norepinephrine by HPLC methods. For 5HT and norepinephrine secretion (basal) and release (stimulated), sister cultures of the hNT2.19 and hNT2.6 cells were differentiated for two weeks before cells were exposed to basal (2.95 mM) or high (100 mM) concentrations of KCl for potassium (K+)-stimulated release for 5HT and norepinephrine measurement in the media. Data represent the mean + SEM from 6–18 samples from >3 independent experiments for each neurotransmitter. Only the hNT2.19 cells contain any 5HT, which is either secreted or released into the extracellular environment; the hNT2.6 cells do not secrete or release 5HT outside the cells. Neither cell line makes, secretes, or releases the neurotransmitter norepinephrine.

The hNT2.19 and hNT2.6 cell lines were differentiated for two weeks in vitro, before HPLC analysis of 5HT and norepi content, basal secretion in the presence of basal K+ (2.95 mM) and stimulated release in the presence of high K+ (100 mM) in the media in the hNT2.19 (Figure 6(a)) and hNT2.6 (Figure 6(b)) cells. The hNT2.19 cell line was able to synthesize significant amounts of the 5HT neurotransmitter, matching the immunohistochemical staining patterns seen above. The norepi content (synthesized) in hNT2.19 was near zero. Mean 5HT content was 485.130 SEM (58.697) pmoles per 10 million cells (*n* = 25). The hNT2.19 cell line also demonstrated significant 5HT release under basal or potassium-stimulated conditions, at the time point during differentiation when these cells were transplanted in the severe contusion SCI pain model. Mean 5HT release under basal (73.381 SEM (16.415) pmoles per 10 million cells, *n* = 21) or stimulated K+ conditions (85.640 SEM (10.515) pmoles per 10 million cells, *n* = 23) over 15 mins was able to account for more than 38% of the total 5HT content in the cell cultures. Mean norepi content (40.154 SEM (10.867) pmoles per 10 million cells, *n* = 13) and secretion (0.0 pmoles per 10 million cells, *n* = 13) or release (0.0 pmoles per 10 million cells, *n* = 13) in the presence of basal and high concentrations of KCl suggested that even though hNT2.19 cells were able to make a very small amount of norepi, they did not release or secrete norepi into the cellular environment. The control hNT2.6 cell line was able to synthesize only very small amounts of the 5HT neurotransmitter, suggested by the lack of 5HT signal in the immunohistochemical staining patterns seen above. The norepi content (synthesized) in hNT2.6 cells was near zero. Mean 5HT content was 78.683 SEM (33.500) pmoles per 10 million cells, *n* = 6. The hNT2.6 cell line also demonstrated no measurable 5HT release under basal or potassium-stimulated conditions, at the time point during differentiation when these cells were transplanted in the severe contusion SCI pain model. Mean 5HT release were zero under basal or stimulated K+ conditions. Mean norepi content, secretion, and release was zero in hNT2.6 in the presence of basal and high concentrations of KCl, which suggested that even though hNT2.6 cells were able to make a very small amount of 5HT, they did not release or secrete 5HT or norepi into the cellular environment.

### 4.2. Characterization of the Grafts of hNT2.19 Cells

#### 4.2.1. Immunohistochemistry of the hNT2.19 Cells after Transplant and SCI

**Figure 7:**
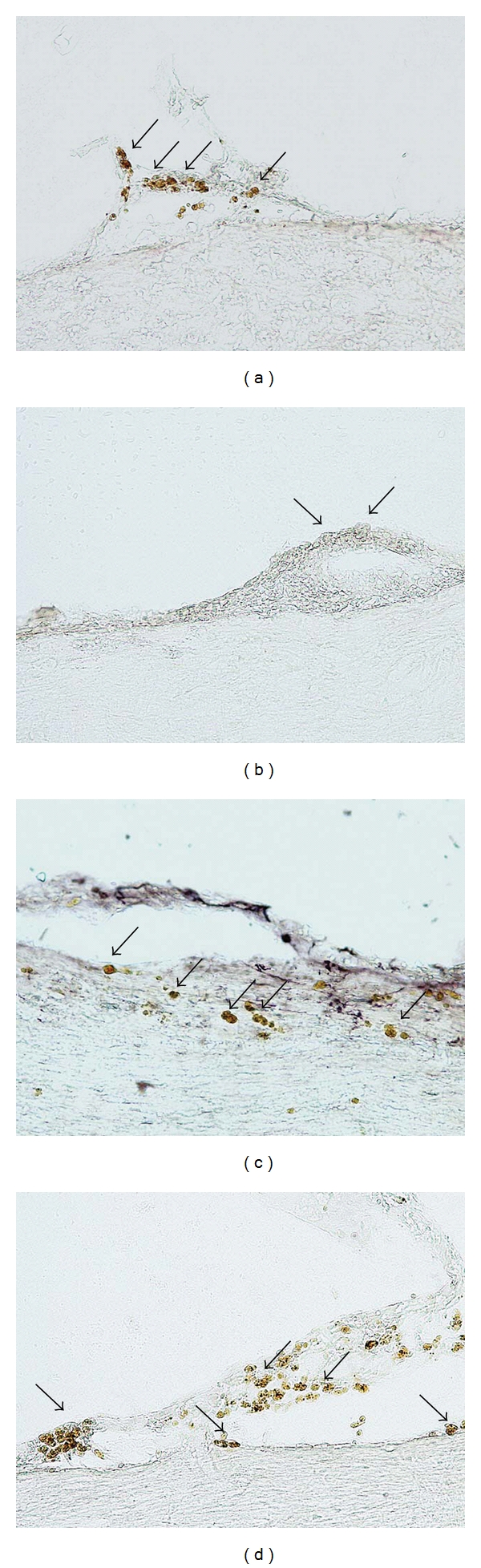
Transplant of hNT2.19 and hNT2.6 cell lines in the severe contusive SCI model: TuJ1 and 5HT immunohistochemistry. Rats were injured with severe contusive SCI followed at two weeks by hNT2.6 (a, b) or hNT2.19 (c, d) cell grafts. Sagittal spinal cord sections were examined at 8 wks after SCI for evidence of surviving lumbar subarachnoid hNT2.6 (a, b) or hNT2.19 (c, d) cell line grafts, utilizing TuJ1 (a, c) or 5HT (b, d) immunohistochemistry. The hNT2.19 and control hNT2.6 (10^6^ cells/injection), which had been differentiated for two weeks in vitro, were injected into the subarachnoid space two weeks after the SCI. Cell graft sites were colocalized with 5HT (b, d) and the human-specific marker TUJ1 (neuron-specific class III *β*-tubulin; (a, c)). There are many surviving hNT2.19 (c) and hNT2.6 (a) grafted cells visible on the pial surface, which stain for TuJ1 (arrows) at the end of the experiment, 56 days after SCI and about 6 weeks after cell transplant. Adjacent sections with the same grafted hNT2.19 (d) and hNT2.6 cells (b) are stained for 5HT, but only the hNT2.19 cells (d) are labeled for 5HT (arrows).

Adult female Wistar Furth rats were injured by contusive SCI induced by the weight drop device and transplanted with intrathecal hNT2.6 (Figures 7(a) and 7(b)) or hNT2.19 (Figures 7(c) and 7(d)) grafts, which had been predifferentiated for two weeks in vitro. Transplant sites (thoracic/lumbar spinal cord) collected 8 weeks after SCI were visualized with specific human and neurotransmitter antibody markers TuJ1 (Figures 7(a) and 7(c)) and 5HT (Figures 7(b) and 7(d)). Many of these grafted cells survive (Figures 7(a) and 7(c), arrows) on the pia near the lumbar cord for at least 8 weeks after SCI, and apparently only the hNT2.19 cells retain their 5HT (compare hNT2.6 cells in Figure 7(b) and hNT2.19 cells in Figure 7(d)) expression after transplant in a severe contusive SCI model.

#### 4.2.2. Sensory Behaviors after Lumbar Subarachnoid Transplant of hNT2.19 Cells

In this study the animals were divided into 6 different experimental groups: (1) Group 1 (Laminectomy); (2) Group 2 (Contusion); (3) Group 3 (Contusion + hNT2 6 cells); (4) Group 4 (Contusion + hNT2 19 cells); (5) Group 5 (Laminectomy + hNT2 19 cells); (6) Group 6 (Laminectomy + hNT2 6 cells). The sensory evaluation included tactile allodynia (TA) and thermal allodynia (TH).

**Figure 8:**
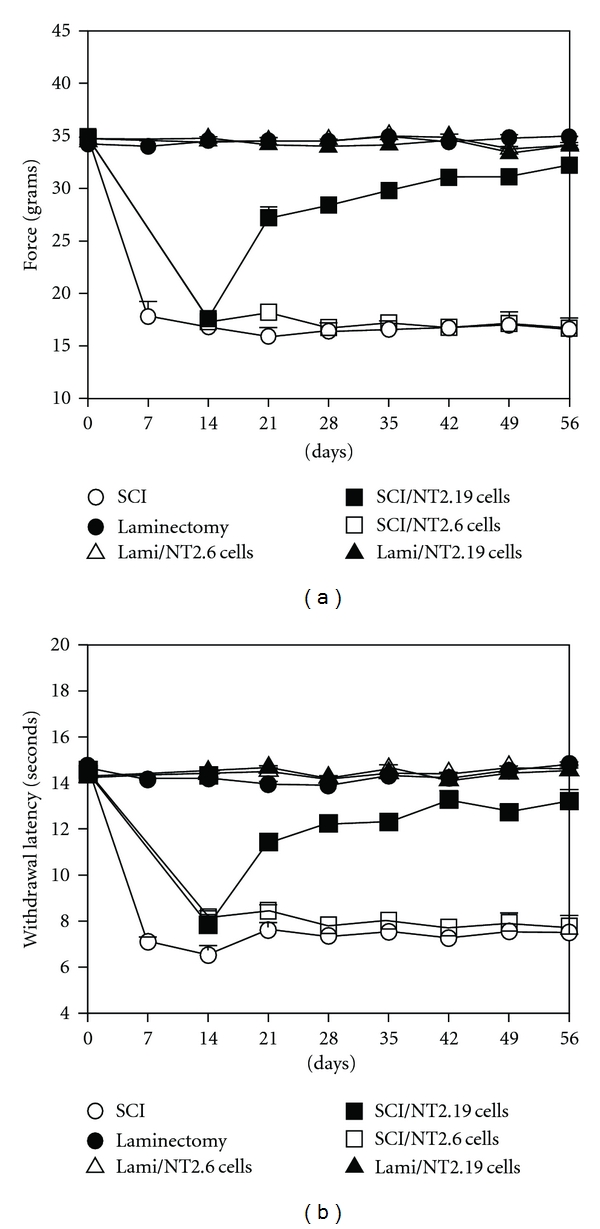
Sensory behaviors after severe contusive SCI and following transplant of hNT2.19 or hNT2.16 cells in vivo. Rats were injured with a weight drop device (NYU impactor, 25 mm) in a rat model of SCI and chronic behavioral hypersensitivity and motor dysfunction. All animals in the study received CsA (10 mg/kg) 1 day before and for 2 wks after the two-week time point (14 days) when some animals were injected with hNT2.19 or hNT2.6 cells. Animals either received SCI alone, laminectomy alone, or SCI plus hNT2.19 or hNT2.6 cells (10^6^ cells/injection), or laminectomy plus either hNT2.19 or hNT2.6 cells into the subarachnoid space at two weeks after SCI. Animals were tested before the SCI (baseline) and once a week following SCI and treatments for hypersensitivity to tactile (a) or thermal (b) stimuli in hindpaws below the SCI. All animals were examined for chronic behavioral hypersensitivity in the right and left hindpaws, but since resultant scores were not significantly different between hindpaws, data was pooled and averaged. SCI injury negatively affected hindpaw responses. Neither hindpaw recovers normal tactile or thermal responses after SCI alone or with transplant of nonserotonergic hNT2.6 cells by 56 days after the severe contusive spinal injury. Data represent the mean value + SEM (*n* = 4–9 animals in each group) at each time point before and 56 days after SCI. Only the hNT2.19 cell grafts attenuated tactile allodynia (a) and thermal hyperalgesia (b) induced by SCI. Recovery of behaviors after graft of hNT2.19 cells was near normal at the completion of the experiments.

In order to provide a basis for the treatment of chronic sensory and motor dysfunction after SCI with neurotransmitter cell therapy, it is critical to examine the effects of hNT2.19 cell therapy on sensory behaviors in a severe weight drop contusive SCI model (Figure 8). Consistent with our previous studies in contusive and other SCI models, evaluating motor and sensory behaviors [49] in combination with cell therapy [23], about one week was required for significant tactile allodynia and thermal hyperalgesia to develop [18]. In our hands, contusive SCI induced significant and bilateral thermal and tactile hypersensitivity in the hindpaws, beginning at about 7 days. The induced sensory abnormalities were not significantly different between the two hindpaws for either TA or TH, and therefore, the ipsi and contralateral sensory thresholds values were averaged. The sensory abnormalities induced by the injury continued without diminution for 60 days, until the animals were sacrificed. Saline (vehicle) injected animals developed no measurable or significant mechanical allodynia or thermal hypersensitivity (data not shown). 

All animals were tested behaviorally at three occasions, one week apart, during two weeks before SCI to establish baseline measures. The behavioral testing continued for 56 days after SCI for TA and TH (Figure 8) as described in Section 2.

**Baseline**

The baseline values were calculated as one average TA and one average TH value. Each average value was based on the ipsi and contralateral threshold values and on the three baseline assessments to increase the robustness of the baseline. ANOVAs (Tables 1(a) and 2(a)) comparing the average TA (*n* = 37; 34.7 SEM 0.9) and the average TH (*n* = 37; 14.48 SEM 0.07) values showed no significant differences among the groups (TA: *F* = 1.03, ns; TH: *F* = 1.46, ns).

**Within-Group Comparisons**

As expected, laminectomy alone (Group 1) or laminectomy followed by cell transplant (Groups 5 and 6) did not have any significant sensory effects over the 60-day period except for Group 1 where TA was slightly higher (*P* < .05) at day 35, and the TH at day 21 and 28 was slightly lower (*P* < .01) compared to baseline, suggesting slight variations between (uninjured) animals (Tables 1(a) and 2(a), Figure 8). However, SCI animals (Group 2) developed significant behavioral hypersensitivity to both thermal and tactile stimuli with significantly (*P* < .001, Bonferroni corrected) lower thresholds on all time points compared to baseline (*t* ranging from −10.66 for *t*7 to −22.09 for *t*14 for TA and *t* ranging from −15.74 for *t*14 to −74.82 for *t*42). After SCI, a significant hypersensitivity to heat was observed about 7 days after SCI that was near maximal at one to two weeks, with mechanical allodynia usually appearing a day or two earlier. The behavioral hypersensitivity responses were not recovered or diminished by 60 days after injection. Transplant of hNT2.19 cells (Group 4) provided permanent attenuation of behavioral hypersensitivity, when transplants were done 14 days after SCI (see Figures 8(a) and 8(b), and Tables 1 and 2).

**Between-Group Comparisons**

The ANOVAs and post hoc comparisons shown in Tables 1(a) and 1(b) Tables 2(a) and 2(b) show that both the TA and TH values differed significantly among groups at all time points after the injury (*P* < 0.000; see Tables 1 and 2) with the greatest differences among groups being 35 days after injury (*F* = 302.5 for TA and *F* = 232.5 for TH). Post hoc analysis (Bonferroni) was used to compensate for multiple comparisons and to show significant differences among groups. Both TA (Figure 8(a) and Tables 1(a) and 1(b)) and TH (Figure 8(b) and Tables 2(a) and 2(b))) were significantly attenuated by the graft of hNT2.19 cells after contusion injury compared to injury alone (*t*21 to *t*56 (*P* < 0.000)) or to graft of nonserotonergic hNT2.6 cells (*t*21 to *t*56 (*P* < 0.000)).

In SCI animals (Group 2), the average mean latency score for TA was 16.8 g (SEM 1.52) at two weeks after SCI. As a contrast, the TA score for the laminectomy control animals (Group 1) was 34.5 g (SEM 0.26) at day 14. This two-week time point after surgery (laminectomy or SCI) was occurring immediately before cell transplants in subsets of those animals, and all injured animals which were to receive cell transplants the next day had mean latency scores not significantly different from injury-alone rats (Group 2 [16.8 g, SEM 1.52] versus Group 4 [SCI/hNT2.19]: 17.60 g), (SEM 0.62), *P* = 1.0. Similarly, the animals with SCI/hNT2.19 cell grafts and SCI/hNT2.6 group [17.03 g (SEM 0.81)] were not significantly different compared to either Group 2 (*P* = 1.0) or Group 3 (*P* = 1.0) at this time point.

Laminectomy rats which were to receive cell transplants the next day [*t* = 14] (Group 5: SCI/hNT2.19 (34.8 g (SEM 0.17))) and Group 6: SCI/hNT2.6 (35.4 g (SEM 0.39)) had mean latency scores not significantly different from laminectomy-alone rats (Group 1 [34.5 g SEM 0.26]), *P* = 1.0, respectively. However, 7 days after the hNT2.19 cells were transplanted near the lumbar spinal cord after SCI (day 21); the threshold for tactile mechanical sensitivity (TA) was significantly (*P* < 0.000) higher (27.2 g; 0.98 (SEM)), compared to both the SCI-alone animals (15.9 g; 0.86 (SEM)) and those receiving the nonserotonin hNT2.6 cell transplants (18.1 g; 0.91(SEM)). The hNT2.19 implants resulted in recovery of 78.8% of the laminectomy-alone value and nearly 30% improvement from day 14 score immediately before transplant for the graft of hNT2.19 cells after SCI. Thus, transplants of the nonserotonin hNT2.6 cells had no significant effect on the development of allodynia by SCI at this time point. By 56 days after SCI, when cell grafts of hNT2.19 had been in place for 6 weeks, the mean threshold value had significantly increased to 32.2 g (SEM 0.47), compared to 17.0 g (SEM 0.50), *P* < 0.000, for SCI alone. At the same time point, 56 days, the mean laminectomy threshold value for Group 1 was 34.9 g (SEM 0.11); as a comparison the laminectomy/hNT2.19 value was 34.0 g(SEM 0.14). These values were not significantly different from each other, *P* = 1.0.

All animals were tested behaviorally at least two weeks before SCI to establish baseline measures and behavioral testing continued for about 60 days after SCI for foot withdrawal in response to noxious thermal (heat) stimulation (thermal hyperalgesia) with a Hargreaves device (Figure 8(b)) as described in Section 2. In laminectomy control animals without SCI (Group 1) there were no significant deviations from baseline with the exception of Day 21 and 28 after laminectomy where withdrawal was slightly but significantly (*P* < .05) faster than at baseline. Similarly, in animals with laminectomy and either hNT2.19 (Group 6) or hNT2.6 transplants (Group 5), no significant differences compared to baseline values were observed in hindlimb withdrawal latency to noxious thermal stimulation over the 60-day period. In SCI animals (Group 2), the mean latency score was 6.53 s (SEM 0.41) at two weeks after SCI (*t*14). At the two-week time point, the score for the laminectomy control (Group 1) animals was 14.2 s (SEM 0.26). This two-week time point after surgery (laminectomy or SCI) was occurring immediately before cells were transplanted in subsets of those animals. All injured animals which were to receive cell transplants the next day (Groups 3 and 4) had mean latency scores of 7.84 s (SEM 0.45); Group 4 (SCI/hNT2.19 and Group 3 [SCI/hNT2.6] had latency scores of 8.17 s (SEM 0.26). These were not significantly different from the latency scores of injury alone rats (Group 2) 6.53 (0.41 SEM) *P* = .19 and *P* = .90. Laminectomy rats which were to receive cell transplants the next day (Group 5 [SCI/hNT2.19]: 14.5 s (SEM 0.12) and (Group 6 [SCI/hNT2.6]: 14.4 s (SEM 0.17) had mean latency scores not significantly different from laminectomy-alone rats (Group 1 [14.2 SEM 0.26]), *P* = 1.0. However, 7 days (Time 21) after hNT2.19 cells were transplanted near the lumbar spinal cord after SCI (Group 4), and the threshold for thermal sensitivity (11.4; SEM 0.39) had improved significantly (*P* < 0.000), compared to both the SCI-alone (7.63 (SEM 0.31) animals and to those receiving the nonserotonin hNT2.6 (8.46; SEM 0.27) cell transplants, although the withdrawal latency was still significantly (*P* < 0.000) shorter compared to Group 1 (14.0; SEM 0.11). This represents 81.8% recovery, compared to laminectomy-alone and greater than 25% improvement after transplant, compared to day 14, immediately before transplant. By 56 days after SCI, when cell grafts of hNT2.19 had been in place for 6 weeks, the mean threshold value had significantly increased to 13.2 (SEM 0.49), which was significantly different *P* < 0.000 compared to 7.50 s (SEM 0.12), for SCI alone. At the same time point (*t*56) the mean laminectomy (Group 1) threshold value was 14.80 s (SEM 0.11); the laminectomy/hNT2.19 (Group 6) value was 14.55 s (SEM 0.10), not significantly different from each other, *P* = 1.0. (see Figure 8(b), Tables 2(a) and 2(b)).

The responses for the various groups were almost identical for SCI alone (Group 2) and SCI plus nonserotonin NT2.6 (Group 3) cells (see Tables 1 and 2, and Figure 8). However, they were significantly different from the responses obtained from the animals with SCI and NT2.19 cell implants. Animals in the latter group recovered near normal sensory responses to tactile (Figure 8(a)) and thermal stimuli (Figure 8(b)), representing 92% and 89% percent recovery, respectively, by 56 days after SCI (6 wks after transplant) after grafting the serotonergic hNT2.19 cells, but not those animals in the SCI-alone group or those in the hNT2.6 cells group.

### 4.3. Motor Behaviors after SCI and Transplant of hNT2.19 or hNT.6 Cells

**Figure 9:**
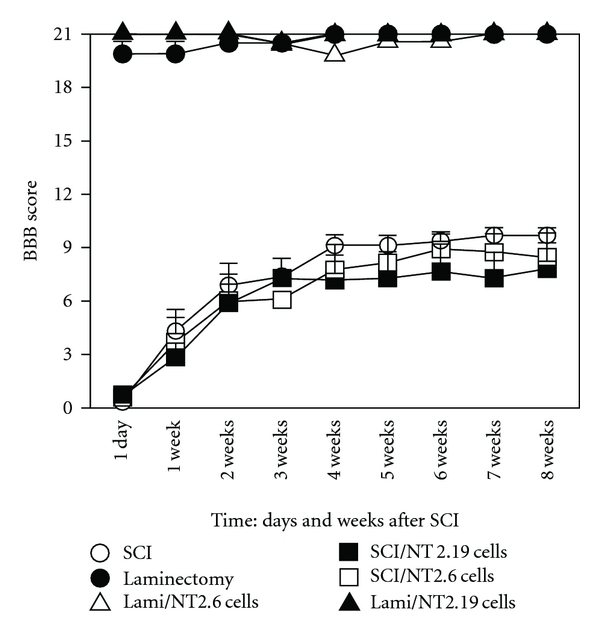
Open-field motor behaviors (BBB) and graft of hNT2.19 or hNT2.6 cells in severe contusive SCI. Gross open-field motor behavioral results show gradual recovery of motor scores beginning at 1 week after SCI, with no additional recovery with the addition of hNT2.19 or hNT2.6 grafts. Data represent the mean value + SEM (*n* > 6 animals in each group) at each time point for 56 days after SCI. BBB scores did not improve over the natural history of recovery after SCI, when either cell line was grafted into the subarachnoid space. Laminectomy alone had no effect on normal BBB scores, and addition of hNT2.19 or hNT2.6 grafted cells to laminectomy animals was not different from laminectomy alone. The hNT2.19 cell therapy, when cells are transplanted into the lumbar subarachnoid space, has no effect on open-field motor behaviors, with or without severe contusive SCI.

Assessment of open-field gross motor behavior using the BBB locomotor scale after SCI (Figure 9 and Table 3) in the presence or absence of either hNT2.19 of hNT2.6 cell grafts revealed that SCI alone, using a 25 mm weight drop injury, resulted in a gradual recovery of motor behavior that reached a plateau at 3-4 weeks following injury and remained essentially unchanged thereafter to the end of the experiment, with the best recovery appearing the last few weeks after SCI. The mean value at 21 days for this group (Group 2, SCI only, *n* = 7) was 7.71 SEM (1.15) and 9.86 SEM (0.46) at 56 days. The decreased BBB scores after SCI were significantly (*P* < 0.000) lower than baseline for all time points. The results for the animals with SCI plus nonserotonin hNT2.6 grafts (Group 3) showed similar decreases that were not significantly different from the animals with contusion only. The BBB scores for the SCI plus hNT2.19 grafts (Group 4) were also nearly identical, with no significant statistical difference from the BBB scores from the SCI plus hNT2.6 or SCI alone animals during all 56 days. Similar comparisons were made between all groups where the ANOVA (Table 3) showed overall significant differences between groups at all time points except for the baseline comparison between the laminectomy-alone group (Group 1, *n* = 5) and neither the laminectomy plus hNT2.6 (Group 6, *n* = 7) or laminectomy plus hNT2.19 (Group 5, *n* = 5) cell graft groups showed significant differences between these groups on any of the time points. The average BBB score at 21 days for this group (Group 1, laminectomy only) was 17.92 SEM (2.62) and 18.92 (2.08) at the experiment's end (56 days). These data suggest that subarachnoid grafts of either hNT2.19 or hNT2.6 cells had no significant effect on the rate or magnitude of limited motor recovery when measured by open-field behaviors for 56 days after severe contusive SCI (see Figure 9 and Table 3). 

**Figure 10:**
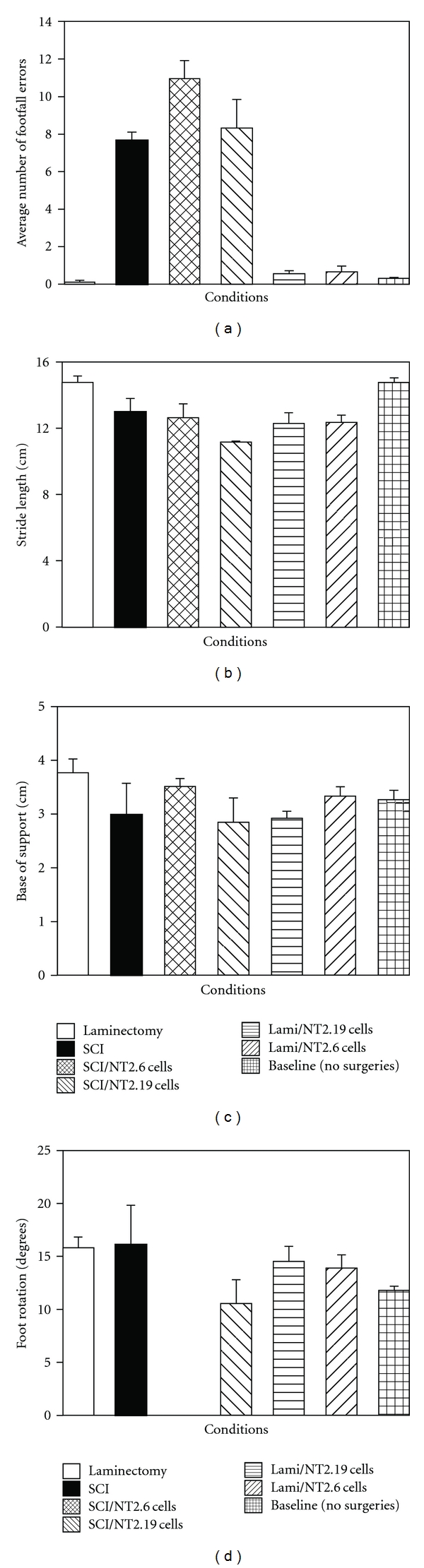
Gridwalk errors/footprint analysis and graft of hNT2.19 or hNT2.6 cells in severe contusive SCI. Gridwalk errors (a), stride length (b), base of support (c), and foot rotation (d) before and at the end of the experiment (day 56) after SCI or laminectomy, with or without transplant of hNT2.19 or hNT2.6 cells at 2 wks after injury. Data show no significant recovery of gridwalk errors, stride length, base of support, or foot rotation with the addition of graft of either cell line, compared to SCI alone. Data represent the mean value + SEM (*n* = 5–8 animals in each group). Laminectomy alone or laminectomy plus either cell line graft had no effect on gridwalk error or footprint scores.

Severe contusive SCI causes a permanent increase in gridwalk footfall errors, decrease in stride length, decrease in base of support, and change in degree of foot rotation in hindlimbs by eight weeks after injury. Initially before injury and at the end of the experiment, all six groups of rats were assessed for footfall error with gridwalk error testing (Figure 10(a)) and footprint analysis of stride length (Figure 10(b)), base of support (Figure 10(c)), and degree of foot rotation (Figure 10(d); day 56). Comparisons between groups, including post hoc tests, showed no significant improvement with the addition of SCI/hNT2.19 or SCI/hT2.6 grafts in gridwalk errors, stride length, base of support, or foot rotation following SCI. The only significant differences observed were between injured and uninjured rats, Groups 2, 3, 4 and Groups 1, 5, 6, respectively, in gridwalk errors and stride length (*F* = 30.913, *P* < 0.000, gridwalk; *F* = 3.102, *P* < .05, stride length; *F* = 1.159, *P* > .05, base of support; and *F* = 0.794, *P* > .05, foot rotation). Footfall error (a) in the SCI rats (Group 2) had a mean value of 7.625 errors SEM (0.449, *n* = 8), while the SCI/hNT2.19 (Group 4) or SCI/hNT2.6 (Group 3) rats had mean values of 8.333 errors SEM (1.522, *n* = 7) and 10.933 errors SEM (0.985, *n* = 5), respectively. The laminectomy (Group 1) or laminectomy plus hNT2.19 (Group 5) or hNT2.6 Group 6) rats had mean values of 0.1 errors SEM (0.1, *n* = 5), 0.533 errors SEM (0.17, *n* = 7) and 0.667 errors SEM (0.291, *n* = 7), respectively. Baseline values (before injury) were 0.305 errors SEM (0.043, *n* = 47). Stride length (b) in the SCI rats (Group 2) had a mean value of 12.974 cm SEM (0.808, *n* = 5), while the SCI/hNT2.19 (Group 4) or SCI/hNT2.6 (Group 3) rats had mean values of 11.15 cm SEM (0.05, *n* = 2) and 12.617 cm SEM (0.835, *n* = 3), respectively. The laminectomy (Group 1) or laminectomy plus hNT2.19 (Group 5) or hNT2.6 (Group 6) had mean values of 14.73 cm SEM (0.404, *n* = 5), 11.15 cm SEM (0.639  *n* = 5) and 12.341 cm SEM (0.436, *n* = 7), respectively, while the SCI/hNT2.19 (Group 4) or SCI/hNT2.6 (Group 3) rats had mean values of 11.15 cm SEM (0.05, *n* = 2), and 12.617 cm SEM (0.835, *n* = 3), respectively. Baseline values for uninjured rats were 14.757 cm SEM (0.269, *n* = 67). Base of support (c) in the SCI (Group 2) rats had a mean value of 2.996 cm SEM (0.571, *n* = 5), while the SCI/hNT2.19 (Group 4) or SCI/hNT2.6 (Group 3) rats had mean values of 2.85 cm SEM (0.45, *n* = 2) and 3.513 cm SEM (0.146, *n* = 3), respectively. The laminectomy (Group 1) or laminectomy plus hNT2.19 (Group 5) or hNT2.6 (Group 6) rats had mean values of 3.766 cm SEM (0.257, *n* = 5), 2.924 cm SEM (0.125, *n* = 5), and 3.339 cm SEM (0.168, *n* = 7), respectively. Baseline values for base of support (uninjured) rats were 3.272 cm SEM (0.169, *n* = 67). Foot rotation (d) in the SCI (Group 2) rats had a mean value of 16.125° SEM (3.708, *n* = 4), while the SCI/hNT2.19 (Group 4) or SCI/hNT2.6 (Group 3) rats had mean values of 10.55° SEM (2.25, *n* = 2) and n.d. (no data), respectively. The laminectomy (Group 1) or laminectomy plus hNT2.19 (Group 5) or hNT2.6 (Group 6) rats had mean values of 15.82° SEM (1.015, *n* = 5), 14.534° SEM (1,428, *n* = 5), and 13.92° SEM (1.219, *n* = 7), respectively. Baseline values for foot rotation (uninjured) rats was 11.804° SEM (0.372, *n* = 67). Inconsistencies in measures, numbers, and missing data, between injured and uninjured rats are related to the severity of injury (25 mm weight drop) causing animals to move poorly by 8 weeks after SCI, even though they have survived SCI and transplant surgeries.

## 5. Discussion

The teratocarcinoma human NT2 (hNT2) parental cell line was the source of the hNT2.19 cell line derived from the embryonic carcinoma (EC) cell type, after differentiation in response to retinoic acid (RA). The NT2 parent cell line has been used for a great variety of studies since its initial description in 1984 [29, 34]. A derivative of the original polyclonal TERA-2 EC cell line, the NT2/D1 line (NT2), is cells with the phenotypic properties of neurons after differentiation, including the expression of neurofilament proteins [50]. This resultant, exclusively neuronal, phenotype with RA treatment has remained a hallmark of this human cell line, unlike other cells of EC origin [51]. The RA-differentiated neurons, called NT2-N cells, are from a committed neuronal cell precursor as determined by lineage analysis [28]. They are similar to developing human spinal cord neurons, reminiscent of terminally differentiated postmitotic neurons. To achieve pure populations of neurons, rapid methods have been developed [52] that include treatment of RA-induced NT2 cultures with mitotic inhibitors to enrich for neurons that express typical neuronal markers [53] with a stable polarized phenotype [52] of central, not peripheral nervous system neurons. A few studies [54] describe a variety of neurotransmitter or neuropeptide phenotypes expressed by NT2-N neurons after 2–4 weeks of differentiation in vitro. The common phenotypes, include 5HT-expressing NT2 cells, range from about 2% [32] to 30% [54], depending on the differentiation procedures. Further increasing the proportion of 5HT producing neurons seems to require particular differentiation protocols involving the timed application of various growth factors [55], methods not used in the current subcloning of the hNT2.19 cell line. Our differentiation method is similar to that of the Guillemain study [54], which provides about 30% 5HT-containing neurons. This explains the relative ease of finding a 5HT-subclone, such as the hNT.19 cell line (see Section 2). A similarly subcloned NT2-derived GABA cell line such as our previously described hNT2.17 cell line, also used in SCI-studies [18], expresses the inhibitory GABA neurotransmitter and simultaneously coexpresses other neurotransmitters such as met-enkephalin or neuropeptide Y [18]. However, the hNT2.19 cell line does not, in our hands, co-express other neurotransmitter markers, such as tyrosine hydroxylase (TH), choline acetyltransferase (ChAT), neuropeptides such as calcitonin gene-related peptide (CGRP), the leu- or met-enkephalins, or neuropeptide Y (NPY). This observation suggests that it is the 5HT secreted by differentiated grafted cells that is the active, antinociceptive agent in this study. Thus, when the hNT2.19 is transplanted in vivo, the grafts apparently retain their 5HT-phenotype. Interestingly, when the parental NT2 parent cell line (which is really a cell population, rather than a phenotype-restricted cell line) is transplanted, a GABA phenotype is favored in vivo [56], but many other phenotypes are possible [57]. Even though multipotentiality of phenotype expression might be an advantage in a clinical use where the therapeutic mechanism-of-action is unknown (i.e., transplant of NT2-N neurons for stroke [31]), a single, more pure phenotype, such as that for 5HT, could be preferable for use in conditions such as neuropathic pain or motor dysfunction following SCI. Graft of 5HT-secreting cells (using a rat cell line) near the spinal cord has been demonstrated to attenuate neuropathic pain after SCI by restoring spinal 5HT and upregulating spinal BDNF, and downregulating the 5HT transporter [8], but effects on the sensory system require a subarachnoid graft location [33], since apparently, in those studies, an intralesion graft site helps restore motor function. We have seen similar results with the use of intraspinal hNT2.19 grafts and the severe contusive SCI model [23]. This “proof-of-concept” and feasibility study makes it clear that graft location for cell therapy approaches in SCI and recovery-of-function should be carefully considered in any transplant strategy, and pain and motor dysfunction might require different graft locations.

Using the rapid aggregation method [35] to differentiate single-cell clones isolated from the NT2 undifferentiated cell line, we were able to identify a number of 5HT-staining cell lines. We chose the hNT2.19 cell line based on its homogeneous morphology and ease of proliferation and differentiation in vitro. Early in the differentiation process, the hNT2.19 cells have multiple neurite extensions (stained for the neurofilament proteins) with medium- to large-size cell bodies, where both cell compartments stain brightly for 5HT. Longer differentiation on the laminin and poly-L-lysine substrate causes them to aggregate into balls, with multiple growing extensions, much like the NT2-N parent cells [28, 54]. They can be lifted and replated without any apparent changes, can be frozen and restarted, and have been maintained without cell division in vitro for greater than 7 weeks. In light of their apparent 5HT phenotype, they consistently maintain features of homogeneous cells: differentiated cells release synthesized 5HT under basal and stimulated in vitro conditions, and apparently not any norepinephrine, which might also be expected to serve as an antinociceptive agent [58].

In spite of the fact that most people who have sustained an SCI develop persistent pain [59, 60], which has profound impact on activities [61] and quality of life after SCI [62, 63], until recently little was known about the mechanisms responsible for this condition. Although pain of musculoskeletal, radicular, visceral, and psychogenic origins play a significant role in the clinical sequelae of spinal injury, central dysesthetic pain is the most disabling and challenging of all sensory complications associated with SCI [64]. While some SCI pain syndromes may respond to therapeutic interventions [65], central neuropathic pain usually fail to respond to any efforts including systemic and local pharmacology [66], neuroaugmentative and neurodestructive approaches [67]. Alteration of the endogenous spinal 5HT system after spinal cord injury (SCI) plays a potential major role in the induction and maintenance of chronic pain in humans. Supraspinal inhibitory pathways that project to the dorsal horn include those that supply the monamines, serotonin (5HT) norepinephrine and dopamine [27, 68–71]. Of these, 5HT is one of the best studied neurotransmitters in SCI [72]. Descending serotonergic pathways originate in brainstem raphe nuclei and terminate in the superficial dorsal and ventral horns of the cord [73, 74], providing excitatory drive to inhibitory systems. Evidence supporting a role for 5HT in antinociception [75–77] is based on its anatomical location, the behavioral effects of intrathecal serotonergic drugs [77–79], and inhibition of spinothalamic tract cells involved in pain transmission [80]. Loss of 5HT acutely after SCI caudal to an injury site is a consistent report [1] in a variety of SCI models including deafferentation [3], spinal hemisection [2, 9], and more recently, clip-compression injuries of the cord [81]. This loss of 5HT after SCI has been used as an indicator of injury severity [82]. After spinal hemisection, with injury-induced tactile allodynia and thermal hyperalgesia, animals develop hypersensitivity to lower doses of intrathecal 5HT for antinociception, related to specific 5HT1A and 5HT3 receptors in the dorsal horn [4]. Grafts of cells that release 5HT into the intrathecal space following dorsal hemisection restore spinal 5HT in the dorsal horn [8], increasing 5HT in the CSF, and correct membrane hyperexcitability and phenotype shifts of dorsal horn neurons [9] associated with tactile allodynia and thermal hyperalgesia following SCI. These same 5HT rat cell line grafts are not antinociceptive when placed within the cord, rather than a subarachnoid location, in the same injury paradigm [33], arguing for a focal application of serotonin to/near the dorsal horn (e.g., the subarachnoid space), without further disturbance to the cord, that is required for an only-antinociceptive strategy with a cell therapy approach.

There is also a significant role for serotonin to enhance motor recovery of function after SCI [72], based on its effects on ventral motor neurons. Endogenous and exogenously applied serotonin modulates the motor system and stimulates motor recovery after SCI [83], with 5HT agonist application seen to directly depolarize *α*-motor neurons [84]. The central pattern generator(s), the local circuitry responsible for rhythmic control of limb movements, is modulated by descending serotonergic inputs [85], where the 5HT spinal innervation is eliminated below the level of SCI following injury. Following spinal transection, rhythmic locomotor function and increased responsiveness to reflex testing can be restored by transplant of embryonic serotonergic raphe cells [86], presumably by replacement of synaptic connections to motor neurons and release of 5HT in the immediate environment [87]. Our earlier published data indicates that, using grafts of rat 5HT cells derived from an immortalized 5HT cell line [5], locomotor function can only be improved after SCI when grafts are placed within the cord; the same grafts effective for nociception are only effective when placed in the subarachnoid space [33], by attenuating the neuronal hyperexcitability induced by SCI in the dorsal horn [9]. The effects on motor neurons by 5HT-secreting intraspinal grafts are presumably by increasing excitability of host neurons through increases in amplitude of monosynaptic reflexes in the central pattern generator circuitry [88], given the excitatory role of 5HT in the ventral horn [83], as opposed to the indirect inhibitory role for 5HT in the dorsal sensory horn system [89]. Agonists for 5HT may facilitate, rather than directly generate, stepping, by enabling the spinal cord neural circuitry to process specific patterns of sensory information associated with weight-bearing stepping, an effect that enhances rehabilitative training [83]. When hNT2.19 are grafted intraspinally, near the contusion site, motor behaviors, such as improvement in open-field movements, fine motor movements, foot rotation, and reduced footfall errors, are improved. Interestingly, these improvements are enhanced when a rehabilitation-like treatment, environmental enrichment, is added to the transplant paradigm [23]. The current study adds further evidence to clarify how human 5HT-secreting neuronal grafts might differently affect sensory and motor recovery after SCI based on graft location, since motor recovery is not affected by subarachnoid grafts of 5HT-secreting cells.

With a likely serotonin-based mechanism to explain the varying effects of the different graft location(s) for the hNT2.19 cell line, it is important to mention how an external source of 5HT might diffuse in the spinal cord environment. And since the hNT2.19 grafts survive well in the subarachnoid space and continue to synthesize the 5HT neurotransmitter in situ, it can be presumed that they function as cellular minipumps to continuously provide 5HT to the immediate environment throughout their survival. Serotonin is known to rapidly degrade metabolically, so a cell-generating continual source might be the best method to provide authentic 5HT, rather than say, a surgical minipump device, as is commonly done in some pain management approaches. But 5HT replacement to or near the spinal cord (such as the dorsal horn sensory system) must take into account the poor diffusion properties of 5HT in the spinal cord [90], where a negative relation between initial injection concentration of 5HT and detection of 5HT with distance from injection site, after intraspinal injection, was seen. With lumbosacral surface applications of 5HT, the amount of 5HT crossing the arachnoid and pia membranes and entering the spinal cord after superfusion was significantly less than that observed with diffusion of 5HT through spinal gray matter after intraspinal microinjection, so that only a relatively low percentage of the applied 5HT is delivered within the cord. The authors report that relatively small amounts of 5HT enter the spinal cord gray matter, with <0.8% of the bath solution concentration being detected in the superficial dorsal horn (within 400 *μ*m of the surface). Even smaller amounts of 5HT reach the intermediate zone and ventral horn. But unlike cell-based sources for 5HT, which continuously secrete and renew the neurotransmitter, a single application of 5HT to the dorsal horn could not be expected to be as effective as a treatment for behavioral hypersensitivity. Whatever barriers to rapid and long-distance diffusion of 5HT exist in the cord, and considering that 5HT entering the dorsal cord would be in much lower concentrations (along a concentration gradient) compared to the same concentration released from a ventral motor cell graft, it is logical to expect a dorsal source not likely to be useful to a ventral motor functionality. The results of the current study support that conclusion.

Important in the consideration of any clinical use of cell line grafts, even those of stem-cell origin [91], the differentiated hNT2.19 cells do not demonstrate any features of a tumor cell line, since they do not express tumor-related genes or is able to incorporate a BrdU signal with differentiation, much like its NT2-N parent [45]. Tumor proteins are abundant in the proliferating hNT2.19 cells, suggesting that only a differentiated hNT2.19 cell would be safe to transplant in vivo. In previous studies [92], we have described the transplant and use of differentiated hNT2 cell lines to treat pain and motor dysfunction after SCI in rats. Grafting well-differentiated hNT2.19 cells into the CNS does not form tumors in rats (in over 100 animals grafted), and their use supports such a contention. 

There is considerable evidence for the use of cell therapy, where grafts function as cellular minipumps in the subarachnoid space in various models of nerve injury [93] and spinal cord injury [94]. For such therapies to reach clinical usage, a number of issues will need to be considered. A typical benefit of such grafts would be the delivery of therapeutic agents, such as neurotransmitters, with a biological half-life too short to be delivered by any other means, (such as the commonly used baclofen mechanical pump used for SCI spasticity) [95–97]. Direct delivery of an endogenous dorsal horn molecule, such as by cell therapy, is a potential approach that we have investigated in the present study. To directly or indirectly supply 5HT or other labile antinociceptive agents via cell therapy is a developing idea in preclinical studies [98]. The next step towards the development of 5HT delivery interventions in human neuropathic pain would include the creation of a human source of expandable 5HT-supplying cells.

Additionally, given the serious outcomes of long-term immunosuppression required in human cell therapy transplants, the issues regarding any required immunosuppression regime for spinal intrathecal cell transplants are complex. Here we have use of a minimal course of CSA (2 weeks following cell transplant) to ensure a modest graft survival in the xenograft model (human to rat). Our earlier studies indicate that at least some time course of CSA is required for adequate graft survival and therapeutic effectiveness [99], representing a minimal use of immunosuppression with a subarachnoid graft in humans. In this earlier study, a number of trends were seen related to immunosuppression regimen: (1) a minimal course of immunosuppression with CsA, about 1 to 2 weeks after transplants, is required; (2) this minimal CsA course ensures optimal efficacy in reversal of the behavioral hypersensitivity associated with SCI pain; (3) less than minimal immunosuppression (1 day) only provides minimal efficacy; (4) longer than the optimal time course of CsA does not improve efficacy significantly. In this study with the similar hNT2.17 grafted cell line, we examined immunostained sections at the end of the experiment and although reliable quantification of grafts is almost impossible, there were clearly fewer surviving grafts with less than 2 weeks of CsA. A “critical” number of functioning grafted cells could influence or permanently affect the therapeutic sensory effects. Precise answers as to possible mechanisms are difficult, but one value of preclinical studies is that they can reveal such differences in outcomes with manipulation of likely clinical variables.

However, it is also important to mention the typical drawbacks that cell therapy for SCI pain might include: (1) possible limits to the achievable levels of a given agent that can be delivered by the cells; (2) possible delivery of a multitude of substances in addition to those of therapeutic interest, many of which cannot be completely defined; (3) dependence on the survival of implanted cells, which may be limited by immunologic factors, nutrient, and oxygen supply, and so forth. Some of these complicating issues for subarachnoid grafts are highly relevant for the future development of potentially beneficial interventions and for the interpretation of the present results including the initial description of the use of a human neuronal hNT2.19 serotonergic cell line graft in an animal model of SCI pain. Hopefully, they will be addressed in later studies with the NT2-derived cell lines.

In summary, cellular therapy for neuropathic pain after severe contusive SCI is a method to chronically deliver potentially antinociceptive molecules, such as 5HT, to the local CNS environment of the spinal cord where the messages for the induction and establishment of chronic pain are initially translated to supraspinal pain centers. Intrathecal transplantation of the hNT2.19 cell line has proven to potently reverse SCI-induced behavioral hypersensitivity (pain-like) behaviors. Genetically modified cell lines can provide a virtually infinite supply of an easily characterized biologic tool to provide analgesia. Such cellular minipumps can be developed as a refined adjunct to the currently used therapies for the management of painful neuropathies.

## Figures and Tables

**Table:** (a)

Time after contusion	Group 1: laminectomy *N* = 5	Group 2: contusion *N* = 7	Group 3: contusion + hNT2-6 *N* = 6	Group 4: contusion + hNT2-19 *N* = 7	Group 5: laminectomy + hNT-19 *N* = 5	Group 6: laminectomy + hNT2-6 *N* = 7	One way ANOVA
	Mean (SEM)	Mean (SEM)	Mean (SEM)	Mean (SEM)	Mean (SEM)	Mean (SEM)	*F*-statistic, *P* value

Baseline	34.2 (0.13)^a^	34.8 (0.24)^b^	34.8 (0.1)^c^	34.8 (0.23)^d^	34.6 (0.16)^e^	34.7 (0.28)^f^	1.03, *P* = .417
7	34.0 (0.51)^a^	17.8 (1.45)^b^	NA	NA	NA	NA	81.9, *P* < .000
14 (10)*	34.5 (0.26)^a^	16.8 (1.52)^b^	17.3 (0.81)^c^	17.6 (0.62)^d^	34.8 (0.17)^e^	35.4 (0.39)^f^	175.9, *P* < .000
21	34.5 (0.28)^a^	15.9 (0.86)^b^	18.1 (0.91)^c^	27.2 (0.98)^d^	34.2 (0.18)^e^	34.5 (0.29)^f^	139.2, *P* < .000
28	34.5 (0.15)^a^	16.4 (0.74)^b^	16.8 (0.61)^c^	28.4 (0.60)^d^	34.0 (0.28)^e^	34.4 (0.27)^f^	264.4, *P* < .000
35	34.9 (0.14)^a^	16.6 (0.79)^b^	17.2 (0.63)^c^	29.8 (0.48)^d^	34.1 (0.10)^e^	35.0 (0.14)^f^	302.5, *P* < .000
42	34.4 (0.17)^a^	16.7 (0.56)^b^	16.8 (0.80)^c^	31.0 (0.59)^d^	34.7 (0.44)^e^	34.8 (0.28)^f^	282.0, *P* < .000
49	34.8 (0.28)^a^	17.0 (0.64)^b^	17.2 (1.09)^c^	31.1 (0.25)^d^	33.4 (0.40)^e^	33.7 (0.27)^f^	217.2, *P* < .000
56	34.9 (0.11)^a^	17.0 (0.50)^b^	16.7 (0.99)^c^	32.2 (0.47)^d^	34.0 (0.14)^e^	34.1 (0.26)^f^	291.8, *P* < .000

*Time after contusion *t*14 is 10 days for groups 2, 3, 4 and 14 days for 1, 6, and 7.

Post hoc analyses (Bonferroni):

^a^Group 1 is significantly different than Groups 2, 3, 4 on all time points.

^b^Group 2 is significantly different than Groups 1, 4, 5, 6 at all time points except for *t*14 when it is not significantly different from group 4.

^c^Group 3 is significantly different than Groups 1, 4, 5, 6 at all time points except for *t*14 when it is not significantly different from group 4.

^d^Group 4 is significantly different from all groups on times 21, 28, 35, 42; *t*14: significantly different from groups 1, 5, 6; *t*49: all but group 5; *t*6: all but 5 and 6.

^e^Group 5 is significantly different from groups 2, 3, 4 on all time points except at *t*49 and *t*56 where group 5 is significantly different only from groups 2 and 3.

^f^Group 6 is significantly different from groups 2, 3, 4 on all time points except at *t*56 where group 5 is significantly different from 2 and 3 only.

**Table:** (b)

Time after surgery	Gr 1					Gr 2				Gr 3			Gr 4		Gr 5
	versus					versus				versus			versus		versus
Groups	2	3	4	5	6	3	4	5	6	4	5	6	5	6	6

TA Baseline	1.0	1.0	0.685	1.0	1.0	1.0	1.0	1.0	1.0	1.0	1.0	1.0	1.0	1.0	1.0
TA 14 (10)*	0.000	0.000	0.000	1.0	1.0	1.0	1.0	0.000	0.000	1.0	0.000	0.000	0.000	0.000	1.0
TA 21	0.000	0.000	0.000	1.0	1.0	0.582	0.000	0.000	0.000	0.000	0.000	0.000	0.000	0.000	1.0
TA 28	0.000	0.000	0.001	1.0	1.0	1.0	0.000	0.000	0.000	0.000	0.000	0.000	0.000	0.000	1.0
TA 35	0.000	0.000	0.000	1.0	1.0	1.0	0.000	0.000	0.000	0.000	0.000	0.000	0.000	0.000	1.0
TA 42	0.000	0.000	0.002	1.0	1.0	1.0	0.000	0.000	0.000	0.000	0.000	0.000	0.001	0.000	1.0
TA 49	0.000	0.000	0.002	1.0	1.0	1.0	0.000	0.000	0.000	0.000	0.000	0.000	0.156	0.027	1.0
TA 56	0.000	0.000	0.018	1.0	1.0	1.0	0.000	0.000	0.000	0.000	0.000	0.000	0.389	0.156	1.0

**Table:** (a)

Time after contusion	Group 1: laminectomy *N* = 5	Group 2: contusion *N* = 7*	Group 3: contusion + hNT2-6 *N* = 6*	Group 4: contusion + hNT2-19 *N* = 7*	Group 5: laminectomy + hNT-19 *N* = 5	Group 6: laminectomy + hNT2-6 *N* = 7	One way ANOVA
	Mean (SEM)	Mean (SEM)	Mean (SEM)	Mean (SEM)	Mean (SEM)	Mean (SEM)	*F*-statistic, *P* value
Baseline	14.6 (0.09)	14.7 (0.14)	14.5 (0.18)	14.5 (0.10)	14.3 (0.12)	14.2 (0.17)	1.46, *P* = .230 (ns)
7	14.2 (0.17)^a^	7.11 (0.21)^b^	NA	NA	NA	NA	607.0, *P* < .000
14 (10)*	14.2 (0.26)^a^	6.53 (0.41)^b^	8.17 (0.26)^c^	7.84 (0.45)^d^	14.5 (0.12)^e^	14.4 (0.17)^f^	144.3, *P* < .000
21	14.0 (0.11)^a^	7.63 (0.31)^b^	8.46 (0.27)^c^	11.4 (0.39)^d^	14.7 (0.10)^e^	14.5 (0.15)^f^	137.3, *P* < .000
28	13.9 (0.13)^a^	7.36 (0.18)^b^	7.80 (0.39)^c^	12.2 (0.26)^d^	14.2 (0.11)^e^	14.2 (0.18)^f^	184.3, *P* < .000
35	14.3 (0.12)^a^	7.54 (0.08)^b^	8.02 (0.38)^c^	12.3 (0.28)^d^	14.6 (0.16)^e^	14.4 (0.09)^f^	232.5, *P* < .000
42	14.2 (0.10)^a^	7.26 (0.08)^b^	7.73 (0.35)^c^	13.3 (0.40)^d^	14.1 (0.30)^e^	14.4 (0.10)^f^	173.8, *P* < .000
49	14.5 (0.20)^a^	7.56 (0.14)^b^	7.89 (0.42)^c^	12.8 (0.29)^d^	14.4 (0.16)^e^	14.6 (0.12)^f^	189.1, *P* < .000
56	14.8 (0.11)^a^	7.50 (0.12)^b^	7.76 (0.49)^c^	13.2 (0.49)^d^	14.6 (0.10)^e^	14.6 (0.18)^f^	120.7, *P* < .000

*Time after contusion *t*14 is 10 days for groups 2, 3, 4 and 14 days for 1, 6, and 7.

Post hoc analyses (Bonferroni):

^a^Group 1 is significantly different than Groups 2, 3, 4 at all time points except for *t*42 when it is not significantly different from group 4.

^b^Group 2 is significantly different than Groups 1, 4, 5, 6 at all time points except for *t*14 when it is not significantly different from group 4 but from group 3.

^c^Group 3 is significantly different than Groups 1, 4, 5, 6 at all time points except for *t*14 when it is not significantly different from group 4 but from group 3.

^d^Group 4 is significantly different from all groups on times 21, 28, 35, 49; *t*14: significantly different from groups 1, 5, 6; *t*42: significantly different from groups 23; *t*56: all but 5 and 6.

^e^Group 5 is significantly different from groups 2, 3, 4 on all time points except at *t*42 and *t*56, where group 5 is significantly different only from groups 2 and 3.

^f^Group 6 is significantly different from groups 2, 3, 4 on all time points except at *t*42 and *t*56 where group 6 is significantly different from groups 2 and 3 only.

**Table:** (b)

Time after surgery	Gr 1					Gr 2				Gr 3			Gr 4		Gr 5
	versus					versus				versus			versus		versus
Groups	2	3	4	5	6	3	4	5	6	4	5	6	5	6	6

TH Baseline	1.0	1.0	1.0	1.0	1.0	1.0	1.0	1.0	0.408	1.0	1.0	1.0	1.0	1.0	1.0
TH 14 (10)*	0.000	0.000	0.000	1.0	1.0	0.19	0.90	0.000	0.000	1.0	0.000	0.000	0.000	0.000	1.0
TH 21	0.000	0.000	0.000	1.0	1.0	0.486	0.000	0.000	0.000	0.000	0.000	0.000	0.000	0.000	1.0
TH 28	0.000	0.000	0.001	1.0	1.0	1.0	0.000	0.000	0.000	0.000	0.000	0.000	0.000	0.000	1.0
TH 35	0.000	0.000	0.000	1.0	1.0	1.0	0.000	0.000	0.000	0.000	0.000	0.000	0.000	0.000	1.0
TH 42	0.000	0.000	0.226	1.0	1.0	1.0	0.000	0.000	0.000	0.000	0.000	0.000	0.448	0.051	1.0
TH 49	0.000	0.000	0.000	1.0	1.0	1.0	0.000	0.000	0.000	0.000	0.000	0.000	0.001	0.000	1.0
TH 56	0.000	0.000	0.028	1.0	1.0	1.0	0.000	0.000	0.000	0.000	0.000	0.000	0.111	1.0	1.0

**Table 3:** ANOVA showing BBB scores.

Time after contusion	Group 1: laminectomy *N* = 6	Group 2: contusion *N* = 7	Group 3: contusion + hNT2-6 *N* = 6	Group 4: contusion + hNT2-19 *N* = 9	Group 5: laminectomy + hNT-19 *N* = 5	Group 6: laminectomy +hNT2-6 *N* = 7	One way ANOVA
	Mean (SEM)	Mean (SEM)	Mean (SEM)	Mean (SEM)	Mean (SEM)	Mean (SEM)	*F*-statistic, *P* value

Baseline	21.0 (0.00)	21.0 (0.00)	21.0 (0.00)	21.0 (0.00)	21.0 (0.00)	21.0 (0.00)	NA
Day 1	16.67 (3.28)	0.29 (0.10)	0.58 (0.58)	0.67 (0.32)	21.0 (0.00)	21.0 (0.00)	73.0; *P* < .000
Week 1	17.25 (2.71)	4.36 (1.39)	3.67 (1.41)	2.83 (0.80)	21.0 (0.00)	21.0 (0.00)	44.6; *P* < .000
Week 2	17.75 (2.78)	7.29 (1.37)	6.00 (1.53)	5.89 (1.07)	21.0 (0.00)	21.0 (0.00)	27.4; *P* < .000
Week 3	17.92 (2.62)	7.71 (1.15)	6.08 (1.58)	7.28 (1.05)	20.4 (1.34)	20.5 (0.50)	24.9; *P* < .000
Week 4	18.83 (2.17)	9.29 (0.65)	7.75 (1.45)	7.17 (1.41)	21.0 (0.00)	19.8 (0.99)	23.6; *P* < .000
Week 5	18.83 (2.17)	9.29 (0.64)	8.17 (1.45)	7.28 (1.50)	21.0 (0.00)	20.6 (0.43)	25.1; *P* < .000
Week 6	18.83 (2.17)	9.57 (0.57)	8.92 (0.85)	7.67 (1.53)	21.0 (0.00)	20.6 (0.43)	25.9; *P* < .000
Week 7	18.92 (2.08)	9.86 (0.46)	8.75 (1.03)	7.28 (1.46)	21.0 (0.00)	21.0 (0.00)	30.3; *P* < .000
Week 8	18.92 (2.08)	9.86 (0.46)	8.50 (1.33)	7.83 (1.45)	21.0 (0.00)	21.0 (0.00)	27.3; *P* < .000

Post hoc analyses (Bonferroni):

Group 1 is significantly different than Groups 2, 3, 4 on all time points.

Group 2 is significantly different than Groups 1, 5, 6 on all time points.

Group 3 is significantly different than Groups 1, 5, 6 at all time points.

Group 4 is significantly different than Groups 1, 5, 6 at all time points.

Group 5 is significantly different than groups 2, 3, 4 on all time points.

Group 6 is significantly different than groups 2, 3, 4 on all time points.
